# Annual Research Review: Associations of socioeconomic status with cognitive function, language ability, and academic achievement in youth: a systematic review of mechanisms and protective factors

**DOI:** 10.1111/jcpp.14082

**Published:** 2024-12-03

**Authors:** Divyangana Rakesh, Paris Anne Lee, Amruta Gaikwad, Katie A. McLaughlin

**Affiliations:** ^1^ Department of Psychology Harvard University Boston MA USA; ^2^ Neuroimaging Department, Institute of Psychology, Psychiatry and Neuroscience King's College London London UK; ^3^ Ballmer Institute University of Oregon Portland Oregon USA

**Keywords:** Childhood and adolescence, socioeconomic status, poverty, cognitive function, language ability, academic achievement, mediators, moderators

## Abstract

Low socioeconomic status (SES) is negatively associated with children's cognitive and academic performance, leading to long‐term educational and economic disparities. In particular, SES is a powerful predictor of executive function (EF), language ability, and academic achievement. Despite extensive research documenting SES‐related differences in these domains, our understanding of the mechanisms underlying these associations and factors that may mitigate these relationships is limited. This systematic review aimed to identify the mediators and moderators in the association of SES with EF, language ability, and academic achievement. Our synthesis revealed stress, support, stimulation, and broader contextual factors at the school‐ and neighborhood level to be important mediators and protective factors in these associations. In particular, cognitive stimulation mediated the association of SES with EF, language ability, and academic achievement. Educational expectations, classroom and school environment, and teacher–student relationships also played a key role in the association of SES with academic achievement. In addition, factors such as preschool attendance, home learning activities, and parental support buffered the association between low SES and lower cognitive and language outcomes. We discuss these findings in the context of interventions that may help to reduce SES‐related cognitive and educational disparities.

## Introduction

The burden of socioeconomic disadvantage is a pervasive issue worldwide (Adamson, 2013; Brooks‐Gunn & Duncan, 1997). Socioeconomic status (SES) is a broad and complex construct that represents access to or possession of both material resources, which is often indexed by income, and non‐material resources such as occupational prestige, educational attainment, and neighborhood quality. Low SES is associated with lower cognitive and academic performance in young people (Smeding, Darnon, Souchal, Toczek‐Capelle, & Butera, 2013; Yeung, King, Nalipay, & Cai, 2022). Further, associations between SES and cognitive and academic outcomes exist across the entire SES distribution and are not confined to children living in poverty (Borgonovi & Pokropek, 2021; Conti, Heckman, & Urzua, 2010; Currie, Shields, & Price, 2007; Noble, McCandliss, & Farah, 2007). These differences in cognitive function and achievement persist throughout childhood and adolescence (Spielberg et al., 2015; Zhang et al., 2020), resulting in disparities in long‐term educational and economic success.

Despite substantial efforts, socioeconomic inequalities in achievement have not declined in recent decades (Chmielewski, 2019). The COVID‐19 pandemic has further highlighted and, in some cases, exacerbated these disparities. Social policy changes during the pandemic, such as shifts in educational delivery and access to support services, have played a role in influencing these trends (Lancker & Parolin, 2020). Our ability to address these disparities has been hampered by a limited understanding of the factors that explain the link between SES and cognitive and academic outcomes, or those that might mitigate these associations. The importance of understanding these mechanisms has been increasingly recognized in recent years (Korous, Causadias, Bradley, Luthar, & Levy, 2022). This review addresses this crucial gap by systematically synthesizing evidence on the mediators and moderators of these links to identify effective intervention targets with the potential to mitigate the impacts of socioeconomic disadvantage on children's long‐term achievement and success.

Early research examined the connections of SES with children's performance on various neurocognitive tasks. These studies demonstrated that some cognitive components are more sensitive to SES than others. Notably, language and executive function (EF) showed stronger associations with SES compared to other cognitive skills (Hackman & Farah, 2009; Merz, Wiltshire, & Noble, 2019; Noble et al., 2007; Noble, Norman, & Farah, 2005). Indeed, SES is particularly related to the cognitive domains of EF and language ability as well as academic achievement (Last, Lawson, Breiner, Steinberg, & Farah, 2018; Merz et al., 2019; Pokropek, Borgonovi, & Jakubowski, 2015; Ribeiro et al., 2023). Positive associations between SES and EF, encompassing constructs such as working memory, attention shifting, and inhibition (Miyake et al., 2000), have been extensively reported (Farah et al., 2006; Last et al., 2018; Lawson, Hook, & Farah, 2018). Indeed, a recent meta‐analysis of 8,760 children from 25 studies found small to medium effect sizes for the association between SES and EF (Lawson et al., 2018). Children's language ability is also strongly influenced by SES, with children from lower SES families exhibiting lower receptive and expressive language ability, phonological awareness, syntax, and vocabulary (Hoff, 2003, 2006; Hoff & Tian, 2005; Letts, Edwards, Sinka, Schaefer, & Gibbons, 2013; Pungello, Iruka, Dotterer, Mills‐Koonce, & Reznick, 2009; Vernon‐Feagans, Garrett‐Peters, Willoughby, & Mills‐Koonce, 2012). Similarly, the association between SES and academic achievement was first shown in a study 600,000 children from across the United States (Coleman, 1968) and is now well‐established (Harwell, Maeda, Bishop, & Xie, 2017; Juan, Peng, & Luo, 2020; Kim, Cho, & Kim, 2019; Korous et al., 2022; Liu, Peng, Zhao, & Luo, 2022; White, 1982).

Evidence from several systematic reviews and meta‐analyses has supported the association of SES with EF, language, and academic outcomes (Harwell et al., 2017; Juan et al., 2020; Kim et al., 2019; Korous et al., 2022; Liu et al., 2022; White, 1982). Importantly however, despite these associations having been investigated extensively, numerous reviews of this literature have highlighted our lack of understanding of the proximal mechanisms underlying the association of SES with cognitive and academic outcomes (Korous et al., 2022; Lawson et al., 2018; Minh, Muhajarine, Janus, Brownell, & Guhn, 2017). While there are a few narrative reviews aimed at discussing these mechanisms (e.g. see Pace, Luo, Hirsh‐Pasek, & Golinkoff, 2017 for a review on pathways linking SES with language outcomes), a recent meta‐analysis of systematic reviews on SES and academic achievement highlighted that future syntheses could significantly advance existing research by investigating the pathways linking SES with cognitive ability and academic achievement (Korous et al., 2022). Our review aims to fill this crucial research gap by conducting a systematic synthesis of the mediators and moderators of the association between SES and EF, language, and academic outcomes to inform future research, interventions, and policy.

While there has been no systematic synthesis of the research on this topic, individual studies have begun to identify potential mechanisms and protective factors (e.g. Dupere, Leventhal, Crosnoe, & Dion, 2010; Raviv, Kessenich, & Morrison, 2004; Sarsour et al., 2011). For example, studies have identified parent mental health issues, family conflict, neighborhood safety, cognitive stimulation – including the quality of the home learning environment, presence of books in the home, language input, and instructional quality at school – and parental warmth and responsiveness, to play an important role in the association between SES and cognitive, language, and academic outcomes (Baydar & Akcinar, 2015; Dilworth‐Bart, 2012; Dulay, Cheung, & McBride, 2018; Gonzalez et al., 2017; Greenfader, 2022; Romeo, Flournoy, McLaughlin, & Lengua, 2022; Suor, Sturge‐Apple, & Skibo, 2017). These factors can be broadly classified into overarching constructs of stressors, social support, and cognitive stimulation. Stressors reflect environmental experiences that are likely to require adaptation and overwhelm the coping resources of an average child (Lazarus & Folkman, 1984; McLaughlin, 2016; Monroe, 2008). Stressors can include events such as family conflict, economic hardship, or exposure to violence, which place substantial demands on a child's coping mechanisms and are well documented to increase risk for a range of physical and mental health challenges (Evans, Li, & Whipple, 2013; Grant et al., 2003; Grant, Compas, Thurm, McMahon, & Gipson, 2004). Cognitive stimulation refers to the availability of enriching environmental inputs that facilitate learning opportunities for children. This includes access to educational materials, intellectually stimulating activities, and an environment that encourages curiosity and cognitive engagement (Rakesh, McLaughlin, Sheridan, Humphreys, & Rosen, 2024). Finally, support is defined as the provision of nurturing relationships, resources, and environments that offer emotional and practical assistance. It encompasses parental warmth, support and responsiveness, supportive and positive relationships with teachers, and a community that provides a sense of belonging and security, all of which can mitigate the adverse effects of stress and promote resilience in children (Chu, Saucier, & Hafner, 2010).

Importantly, in addition to these proximal factors, Bronfenbrenner's ecological systems theory (Bronfenbrenner, 1979) highlights the influence of individual characteristics, such as temperament and broader contextual factors, including neighborhood and school environments on developmental outcomes. Child characteristics – encompassing cognitive and language abilities, personality, and temperament (Beisly, Kwon, Jeon, & Lim, 2020; Greenfader, 2022; Kriegbaum & Spinath, 2016; Nesbitt, Baker‐Ward, & Willoughby, 2013; Sun, Zhang, Chen, Lau, & Rao, 2018; Zhang et al., 2013), – as well as neighborhood‐level exposures, such as social organization and the physical environment (Lei, 2018), also mediate the association of SES with academic outcomes. Our systematic review will therefore classify factors into proximal environmental factors (stress, support, and stimulation), children's characteristics, and other contextual factors (neighborhood and school characteristics), with any factors not falling into these categories examined separately (see Table 1 for a comprehensive definition).

**Table 1:** Definitions of different categories

Category	Definition
Stress	Stressors reflect environmental experiences that are likely to require adaptation and overwhelm the coping resources of an average child. Stress in this study encompasses the broad range of environmental and interpersonal challenges that demand significant adaptation from a child, contributing to psychological and emotional strain. This includes economic hardships, family instability, exposure to violence, and unpredictable environments. Additionally, stress arises from negative interpersonal dynamics within the family and school settings, as well as from significant life events that disrupt a child's sense of security. Overall, stressors are those that impact the child's well‐being and development, requiring them to adapt and cope with adverse conditions
Support	Support is defined as the provision of nurturing relationships, resources, and environments that offer emotional and practical assistance. This encompasses the emotional climate within the family, the responsiveness and sensitivity of caregivers, and the quality of interactions between children and their teachers. Supportive environments are characterized by warmth, acceptance, and consistent engagement, both within the home and the broader community. Variables include parental support, such as maternal and paternal responsiveness, emotional involvement, and warmth, as well as parental encouragement of maturity and positive interactions, which together create a nurturing and accepting environment. Teacher support involves emotional support and positive interactions that foster a supportive teacher–student relationship. Additionally, community support includes assistance from neighbors and access to social resources, contributing to a holistic support system that enhances children's emotional security and developmental outcomes
Stimulation	The availability of enriching environmental inputs that facilitate learning opportunities for children. This includes access to learning materials such as books and educational resources, as well as engaging in cognitively stimulating activities like shared reading, storytelling, and educational play. Caregiver involvement plays a crucial role through activities such as parental education investment, discussions of school‐related issues, and support for homework. Additionally, linguistic stimulation from caregiver speech and vocabulary, as well as the overall quality of the home learning environment, contributes significantly to a child's development. The presence of organized activities, cultural resources, and outdoor opportunities for learning further enhance the child's exposure to stimulating experiences, promoting intellectual growth and academic achievement
Child characteristics	Individual attributes and abilities of children that influence their cognitive, emotional, and academic development. These characteristics include emotional traits such as negative and positive affect and temperament, and other factors such a bilingualism
Other contextual factors	Other contextual factors refer to broader environmental elements that impact children's development, including the physical and institutional resources of neighborhoods (such as facilities and extracurricular activities), neighborhood social organization, and the physical environment. It also includes school‐related factors like the classroom environment, school resources, and school climate. These factors differ from those under stress, support, and stimulation by focusing on the structural and institutional attributes of neighborhoods and schools rather than the immediate emotional support, practical assistance, or enriching inputs experienced by the child
Other	Factors that did not fall into any of the above categories

This review offers a novel contribution to the literature by providing a comprehensive synthesis of the mediators and moderators influencing the relationship between SES and EF, language, and academic outcomes. Unlike narrative reviews, which often provide broad overviews without systematically evaluating evidence, this systematic review rigorously analyzes the extant literature to identify consistently implicated factors and mechanisms, to help identify maximally effective intervention targets. By identifying modifiable factors through this synthesis, we aim to provide actionable insights that could help mitigate socioeconomic disparities in children's developmental outcomes.

## Methods

### Literature search and screening

Our systematic review followed the PRISMA guidelines (Moher, Liberati, Tetzlaff, & Altman, 2009). To identify relevant studies, in line with previous meta‐analyses in the area (Lawson et al., 2018), we performed literature searches in the two biggest databases for psychology and education research: ERIC and PsycInfo. The search was conducted on September 19, 2022, without any restrictions on the publication date (re‐run in February 2024; see below). Our search included terms related to SES, age groups (including childhood, child, youth, and adolescence), EF, language, and academic achievement (e.g. executive function, language, verbal fluency, academic performance, academic achievement), as well as terms related to mediation and moderation (e.g. mediate, moderate, mechanism, buffer). SES terms were the same as our previous systematic review on the neural correlates of SES (Rakesh & Whittle, 2021) and covered a range of words including socioeconomic disadvantage, socioeconomic status, poverty, neighborhood disadvantage, neighborhood SES, SES, neighborhood, household income, parent income, disadvantage, socioeconomic factors, Hollingshead, and parent education. Terms for EF were the same as a recent meta‐analysis on the topic (Lawson et al., 2018) and covered a range of terms related to EF (i.e. executive function, cognitive control, executive functioning, self‐regulation, working memory, inhibition, inhibitory control, shifting, cognitive flexibility, attention). Our search yielded 3,633 unique titles and abstracts after deduplication.

**Figure 1:**
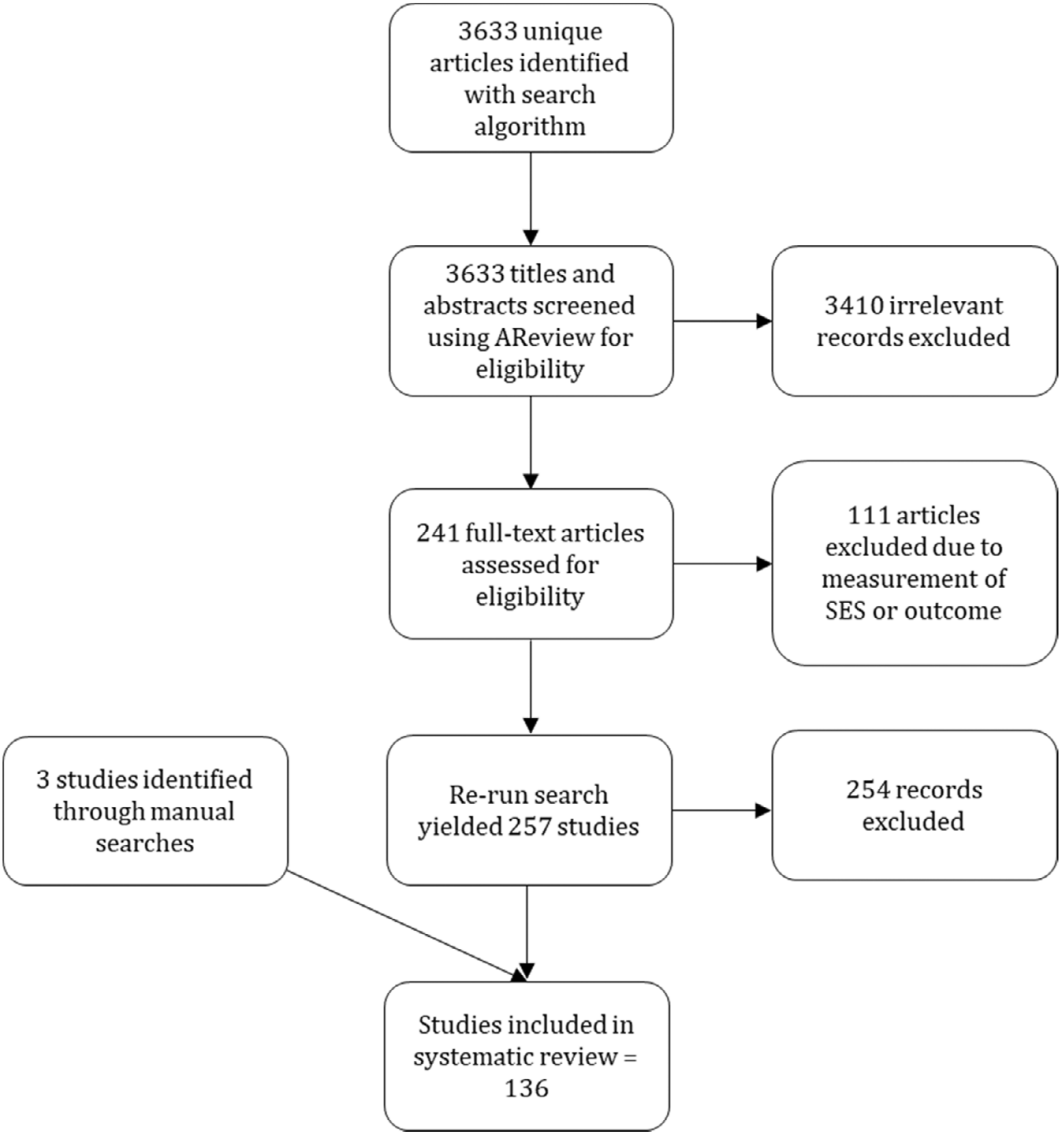
PRISMA diagram

We screened all titles and abstracts using a tool called Active learning for Systematic Reviews or ASReview, which is a machine‐learning‐based tool to conduct systematic reviews (van de Schoot et al., 2021). ASReview uses a combination of human input and machine learning to iteratively select and classify relevant research papers for inclusion in a systematic review. It starts with a small set of researcher‐labeled records, and then uses active learning to suggest the most informative papers for further review by the researcher. Starting from a randomly presented list of records, the researcher labels the record as ‘relevant’ or ‘not relevant’. ASReview learns from the researchers' input and progressively reorders the records from most to least relevant after every decision based on the machine learning algorithm. This iterative process continues until a predetermined stopping criterion is met. Simulations indicate that screening a range of 8%–33% of records can identify at least 95% of the relevant records (van de Schoot et al., 2021). Based on this recommendation and taking a conservative approach, we determined that we would screen at least 40% of the records and only stop if no records were identified as relevant for at least 150 items. We opted for ASReview to mitigate bias and streamline the review process. However, it is important to acknowledge that ASReview is not entirely bias‐free and can only be effective when the selection criteria and stopping rule are pre‐established. For further details on ASReview, refer to van de Schoot et al. (2021). Initially, one author screened the titles and abstracts using ASReview. A total of 2062 papers (56% of the total) were screened using ASReview before reaching the stopping rule by one author. An additional author screened all items marked as irrelevant to confirm ineligibility. Subsequently, at the full‐text review stage, two authors assessed the eligibility of the remaining papers (*n* = 241; ~6% of 3,633). Any discrepancies were resolved through discussion, ultimately reaching a consensus with 100% agreement. One hundred and thirty studies met inclusion criteria and were included in the review. To include recently published studies, we reran the search on 8th February 2024. This search yielded 257 new items, which were screened manually. Sixteen items (~6%) were identified as relevant for full‐text review, of which three met inclusion criteria. The same approximate percentage of articles being identified for full text review through ASReview and manual screening reinforces the credibility of ASReview. Three additional articles were identified through manual searches. Ultimately, 136 articles satisfied our inclusion criteria and were included in our systematic review (see Figure 1 for a PRISMA diagram).

### Inclusion and exclusion criteria

Studies were included if they: (a) examined associations between SES and at least one measure of a behavioral task of EF and/or standardized/behavioral assessments of language or academic performance in children and adolescents, and (b) examined at least one moderator or mediator of these associations. Since behavioral measures and ratings of EF are not highly correlated and appear to assess different constructs (Toplak, West, & Stanovich, 2013), our review focused on behavioral measures of EF rather than parent or teacher‐reported ratings. This approach provides an objective measure of EF that is free from variation based on the child's behavior in different contexts (e.g. home vs. school) and the rater's perspective and is consistent with prior meta‐analyses on SES and EF (Lawson et al., 2018). To make comparisons across studies, academic performance had to be measured using standardized tests (e.g. Woodcock Johnson) as opposed to teacher or student reported grades on tests that can vary substantially in difficulty based on the school and teacher. Studies were included if they used objective (as opposed to perceived) financial (e.g. income, reduced‐price lunch status), non‐financial (educational attainment, occupational prestige, neighborhood poverty, preschool, or school SES), or composite (e.g. Hollinghead index) measures of SES at the household, neighborhood or school level, and they had participants in the age range of childhood to late adolescence (mean age ≤18 years) with an upper age limit of 24 years. The age range was based on recent recommendations regarding the adolescent period ranging from 10 to 24 (Sawyer, Azzopardi, Wickremarathne, & Patton, 2018). Included studies had to be peer reviewed articles published in English. Quality assessment was conducted using the NIH Quality Assessment Tool for Cohort and Cross‐Sectional Studies. Most studies were classified as ‘Good’ (109; 80%), with the rest rated as ‘Fair’.

### Data extraction and synthesis

For each study, we extracted the following information: (a) participant mean age and age range, (b) number of participants, (c) SES measure used, (d) EF, language, or academic achievement measure, (e) moderator or mediator variable, and v(f) key findings. Findings of interest included results from (a) models where interactions between SES and a protective or risk factor predicted EF, language, or academic outcomes, and (c) mediation models examining the indirect effect of a putative mediator in the association of SES with EF, language, or academic outcomes. To synthesize findings, we first grouped studies by outcome (i.e. EF, language performance, and academic achievement). Next, we grouped findings based on the type of analysis run – mediation versus moderation (see Table 2). Finally, within mediation and moderation studies, we grouped studies by the type of mediator/moderator examined. Types included: stress, support, stimulation, neighborhood and school‐level factors, child characteristics, and other factors (which included those that did not belong in any of the above categories). Due to a limited number of studies examining individual SES measures for each mediator and outcome, results were combined across SES indices. Of note, cognitive and language variables considered as outcomes in this review were also considered as mediators/moderators of the association of SES with academic achievement. Where possible, we provide the percentage of studies that report significant mediation or moderation. For mediation, where findings were impacted, we additionally comment on whether the results changed when only studies with temporal precedence (where the exposure and mediator were assessed prior to the outcome or where all three variables were temporally separated) were included in the synthesis. Notably, in most cases, the percentages did not change substantially under this criterion. Details of individual studies have been provided in Supporting Information (Tables S1–S6). Further, mediators that did not belong in the stress, support, or stimulation categories and were examined in a single study are not described in detail in text but are available in the Supporting Information.

**Table 2:** Number of studies in each category

Outcome	Mediation	Moderation
Executive function	17	13
Language	25	4
Academic achievement	68	25

## Results

Across studies, the categories of mediators and moderators were divided into five groups: stress, stimulation, support, child characteristics, and other contextual factors. Studies were classified as ‘other’ if they did not fall into any of the above categories.

### Executive function

#### Mediation

##### Stress

**Figure 2:**
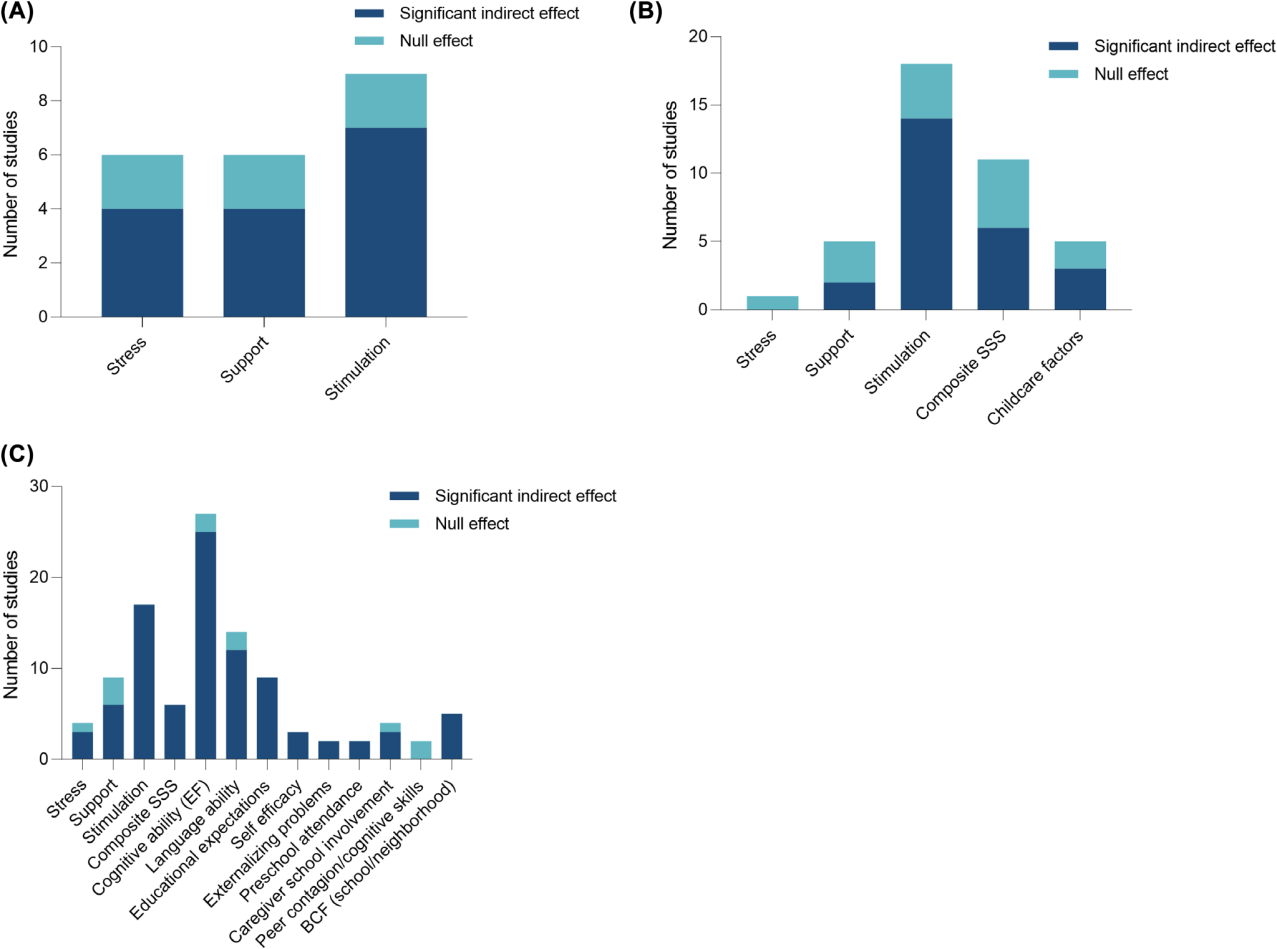
Findings for mediators of the association of SES with executive function (A), language ability (B), and academic achievement (C). Number of studies with at least significant indirect effect (dark blue) versus studies reporting no significant indirect effects (teal) for each category of mediator. Composite SSS = composite measures of the environment that include stress, support, and stimulation and BCF are other contextual factors including schools and neighborhoods

Four of seven (57%) studies found that stress – including harsh discipline, parent stress, self‐reported stress, and financial strain – mediated the association between low SES and lower EF (Baker, Huang, Liu, & Battista, 2021; Encinger, Shizu Kutaka, Chernyavskiy, Acar, & Raikes, 2020; He & Yin, 2016; Suor et al., 2017). See Figure 2A. However, findings were somewhat mixed for individual measures of stress. For example, of the two studies that examined parental stress, only one study (50%) found it to be a mediator between SES and EF (Encinger et al., 2020). Indicators of stress that did not significantly mediate this association included stressful life events, adolescent stress, threat, and harsh parenting (Hackman, Gallop, Evans, & Farah, 2015; Vogel, Perry, Brandes‐Aitken, Braren, & Blair, 2021; Vrantsidis, Clark, Chevalier, Espy, & Wiebe, 2020). Importantly, if only studies with temporal precedence (where the exposure and mediator were assessed prior to the outcome or where all three variables were temporally separated) were included in the synthesis, only one study (1/4; 25%) reported significant mediation results (Suor et al., 2017).

##### Stimulation

Stimulation, including cognitive stimulation, linguistic input, and home enrichment, was found to significantly mediate the association between SES and EF in seven of nine studies (78%) (Daneri et al., 2019; Dilworth‐Bart, Poehlmann, Hilgendorf, Miller, & Lambert, 2010; Hackman et al., 2015; Lipina et al., 2013; McCoy, Zuilkowski, & Fink, 2015; Rosen, Meltzoff, Sheridan, & McLaughlin, 2019; Sarsour et al., 2011). However, findings were mixed at the level of individual indicators. For example, studies found both significant and non‐significant indirect effects of overall cognitive stimulation (McCoy, Zuilkowski, & Fink, 2015; Rosen et al., 2019; Vrantsidis et al., 2020; Wolf & McCoy, 2019). If only studies with temporal precedence were included in the synthesis, four of seven studies reported significant mediation (57%).

##### Support

Support – including maternal warmth and sensitivity, family companionship, and maternal emotional and attentional scaffolding – mediated the positive association between SES and EF in four of six studies (67%) (Dilworth‐Bart et al., 2010; Hackman et al., 2015; Sarsour et al., 2011; Yu, O'Brien Caughy, Smith, Oshri, & Tresch, 2020). However, some indicators of support such as maternal emotional scaffolding and responsiveness, teacher emotional support, and family companionship and integration did not mediate these associations (Dilworth‐Bart et al., 2010; Hackman et al., 2015; Sarsour et al., 2011; Wei, McCoy, Busby, Hanno, & Sabol, 2021; Zhao, Cao, & Maes, 2023). In addition, aggregate measures of stress, support, and stimulation, such as maternal psychological distress, overall quality of the home environment, and overall measure of deprivation, mediated the association of SES with EF (Sarsour et al., 2011; Vogel et al., 2021; Vrantsidis et al., 2020). Findings did not change substantially under the temporal precedence criterion.

##### Other factors

Years of early childhood education also mediated the association of SES with EF (McCoy, Zuilkowski, & Fink, 2015). Several other factors, such as instructional support, maternal behavioral and intellectual involvement in schoolwork, classroom organization, caregiver school involvement, birthweight, gestational age, and negative affect were investigated in one study each (Hackman et al., 2015; He & Yin, 2016; Wei et al., 2021; Wolf & McCoy, 2019; Zhao et al., 2023). See Table S1 for details.

#### Moderation

##### Stress, support, and stimulation

**Figure 3:**
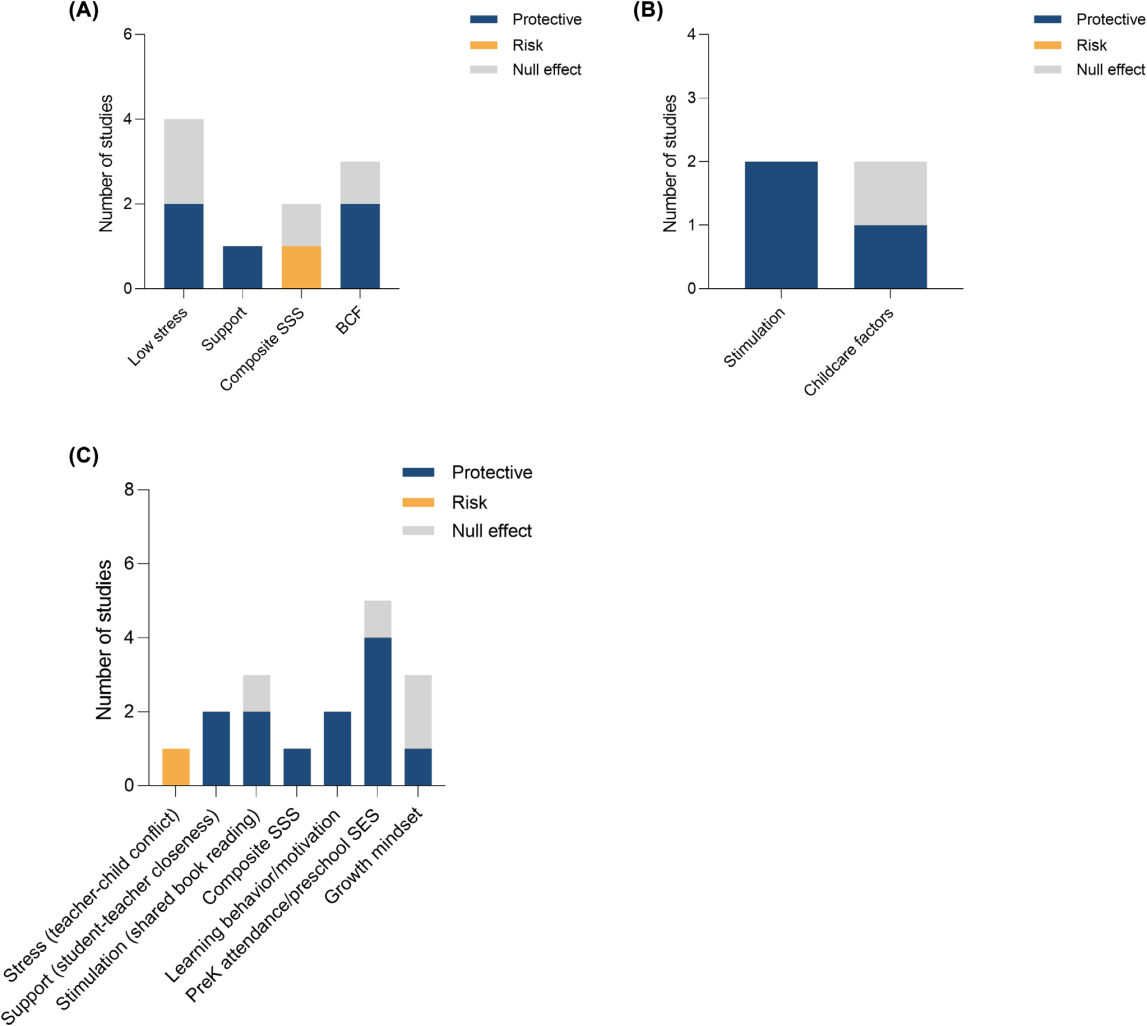
Findings for moderators of the association of SES with executive function (A), language (B), and academic achievement (C). Number of studies that found a protective effect versus increased risk. A protective effect is one that reduces the association between low SES and lower academic achievement and a risk effect is one that strengthens it. SSS = composite measures that capture stress, support, and stimulation (the risk effect was observed for single‐parent status). BCF = broader contextual factors in the form of academic support and neighborhood resources

Of the four studies that examined stress (Baker et al., 2021; Ming, Zhang, Jiang, Ren, & Huang, 2021; Rochette & Bernier, 2014; St. John & Tarullo, 2020), two studies (50%) found lower levels of stress, in the form of low subjective financial strain and high subjective SES to buffer the association between low SES and lower EF (Baker et al., 2021; Ming et al., 2021). See Figure 3A. However, household chaos and maternal hostility/rejection did not (Rochette & Bernier, 2014; St. John & Tarullo, 2020). Support, in the form of maternal responsiveness and physical proximity attenuated the association between low SES and lower EF in one study (Rochette & Bernier, 2014). Of parental well‐being and single‐parent status, both of which capture aspects of stress, support, and stimulation, only single‐parent status moderated the association between SES and EF. Specifically, children from single‐parent families performed less well relative to children from two‐parent families of similar SES (Sarsour et al., 2011), which suggests that coming from a two‐parent family may buffer risk for lower EF among low‐SES youth.

##### Child‐characteristics

Lower levels of temperamental reactivity (Raver, Blair, & Willoughby, 2013) and bilingualism (Hartanto, Toh, & Yang, 2019) reduced the strength of the association between low SES and lower EF.

##### Broader contextual factors

SES was more positively associated with inhibitory control and working memory at higher levels of neighborhood ‘chaos’ (St. John & Tarullo, 2020). Findings were mixed for neighborhood resources, with one study finding a protective effect (Wei et al., 2021) and another reporting a null finding (Cubides‐Mateus, LoCasale‐Crouch, & Turnbull, 2023). School level academic support attenuated the association between low neighborhood SES and lower EF (Piccolo, Merz, & Noble, 2019).

##### Other factors

Parent EF was associated with child EF, and high levels of TV viewing had a negative association with EF, only in low‐income households (Kao, Nayak, Doan, & Tarullo, 2018; Ribner, Fitzpatrick, & Blair, 2017). This suggests that high parent EF and low TV watching may buffer the association between low SES and low child EF. In addition, teacher stress moderated the association between school‐level SES and EF such that when teachers reported high levels of stress, there was no significant difference in child EF performance between high‐ and low‐poverty schools. However, low teacher stress benefited child EF in low‐poverty schools but had the opposite effect in high‐poverty schools (Neuenschwander, Friedman‐Krauss, Raver, & Blair, 2017). Sleep efficiency (Philbrook, Hinnant, Elmore‐Staton, Buckhalt, & El‐Sheikh, 2017) and subjective social mobility (Ming et al., 2021) did not moderate associations between SES and EF. See Table S2 for details.

### Language

#### Mediation

##### Stress

Stress, in the form of negative teacher–child interactions, was not found to mediate the association between SES and language ability (McCoy, Connors, Morris, Yoshikawa, & Friedman‐Krauss, 2015). No other studies examined stress as a mediator. See Figure 2B.

##### Stimulation

In 14 of 18 studies (78%), stimulation mediated the positive association between SES and language outcomes (Baydar & Akcinar, 2015; Cheung & Wong, 2021; Coddington, Mistry, & Bailey, 2014; Foster, Lambert, Abbott‐Shim, McCarty, & Franze, 2005; Gonzalez et al., 2017; Hoff, 2003; Iruka, Dotterer, & Pungello, 2014; Lurie et al., 2021; McCoy, Zuilkowski, & Fink, 2015; McNally, McCrory, Quigley, & Murray, 2019; Mendive, Lissi, Bakeman, & Reyes, 2017; Raviv et al., 2004; Singh, Yeung, Cheng, & Heng, 2023; Swanson et al., 2019). These mediators included variables that captured overall home stimulation as well as access to learning materials and books, language exposure, reading activities and frequency, parent reading beliefs, parent education investment, caregiver–child interactions, and outdoor activities. However, studies also reported null results for similar variables that capture stimulation such as home literacy activities, shared reading, language exposure, and expressive communication. Findings did not change substantially under the temporal precedence criterion (8/11; 73%).

##### Support

Support – including maternal responsiveness and sensitivity – mediated the positive association between SES and language outcomes in two of five studies (40%) (Baydar & Akcinar, 2015; Raviv et al., 2004), with some mixed results; two studies (67%) found maternal responsiveness and sensitivity to be a significant mediator of this association, while another (33%) reported null results for responsive parenting (Baydar & Akcinar, 2015; Loboda, Vogelbacher, & Gawlitzek, 2017; Raviv et al., 2004). Other indicators of support including support from and positive interactions with teachers and support from neighbors did not mediate the association between SES and language outcomes (Baydar & Akcinar, 2015; McCoy, Connors, et al., 2015; Wei et al., 2021). Under the temporal precedence criterion, no studies reported significant mediation.

In addition, six of 11 studies (55%) assessing aggregate measures of stress, stimulation, and support found early childhood environment, parenting style – including authoritative and inconsistent parenting – and quality of the home environment to be significant mediators of the positive association between SES and language outcomes (Cheung & Wong, 2021; Coddington et al., 2014; Dupere et al., 2010; Kohen, Leventhal, Dahinten, & McIntosh, 2008; Loboda et al., 2017; Rubio‐Codina, Attanasio, & Grantham‐McGregor, 2016). However, results were sometimes mixed for parenting style; parental authoritativeness and inconsistent parenting, but not assertive, demanding, or punitive parenting, mediated the association between SES and language ability (Baydar & Akcinar, 2015; Cheung & Wong, 2021; Kohen et al., 2008; Loboda et al., 2017). Parent self‐efficacy, non‐present talk, parent depression, single parenthood, and social risk (operationalized as a composite of parent psychopathology, violence, social isolation) did not mediate these associations (Dulay et al., 2018; Dupere et al., 2010; Foster et al., 2005; McNally et al., 2019; Mendive et al., 2017). Findings did not change substantially under the temporal precedence criterion (4/9; 44%).

##### Broader contextual factors

Classroom environment, organization, and quality, school advantage, and physical resources in the neighborhood did not mediate the association between SES and language ability (Baydar & Akcinar, 2015; Dupere et al., 2010; McCoy, Connors, et al., 2015).

##### Other

Three of five studies (60%) found factors associated with child care (i.e. attendance, quality) to mediate the association of SES with language outcomes (Dulay et al., 2018; Dupere et al., 2010; McCoy, Zuilkowski, & Fink, 2015). Findings did not change substantially under the temporal precedence criterion. Several other factors, such as duration of breastfeeding, first‐born child status, planned pregnancy, and birth after 36 weeks of gestation, instructional quality, peer cognitive skills, instructional support, height‐for‐age, family size, and teacher expectations were investigated in one study each (see Table S3 for more information).

#### Moderation

Four studies examined moderators of the association between SES and language ability and found that full time nonmaternal care, maternal referential question asking, and maternal responsiveness were associated with better language outcomes among children from low‐ and/or middle‐SES households but not high‐SES households (Baydar & Akcinar, 2015; Geoffroy et al., 2007; Luo, Masek, Alper, & Hirsh‐Pasek, 2022). This suggests that full‐time non‐maternal care and maternal referential question asking (in the first 2 years of a child's life) may buffer the association of low SES with language outcomes. In addition, the language outcomes of low‐SES children may benefit more from maternal responsiveness. See Table S4 and Figure 3B for details.

### Academic achievement

#### Mediation

##### Stress

Three of four studies (75%) that examined stressors – including parental stress, material hardship, and household instability – as mediators observed at least one significant indirect effect (Chien & Mistry, 2013; Crosnoe & Cooper, 2010; Garrett‐Peters, Mokrova, Vernon‐Feagans, Willoughby, & Pan, 2016). See Figure 2C.

##### Stimulation

Stimulation mediated the association between SES and academic achievement in all 17 studies that investigated it (100%) (Altschul, 2012; Baker, Kainz, & Reynolds, 2018; Betancur, Votruba‐Drzal, & Schunn, 2018; Bodovski & Farkas, 2008; Carolan, 2016; Chien & Mistry, 2013; Crosnoe & Cooper, 2010; Eamon, 2002; Forget‐Dubois et al., 2009; Galindo & Sonnenschein, 2015; Hamilton, Hayiou‐Thomas, Hulme, & Snowling, 2016; Iruka et al., 2014; Larson, Russ, Nelson, Olson, & Halfon, 2015; Marks, Cresswell, & Ainley, 2006; Myrberg & Rosén, 2008, 2009; Wolf & McCoy, 2019). Higher levels of cognitive stimulation – including the number of books in the household, availability of learning and cognitively stimulating materials, participation in organized activities, and early reading activities – mediated the positive association between SES and academic achievement. Findings did not change under the temporal precedence criterion (14/14; 100%).

##### Support

Six of nine studies (67%) reported that support – including social resources, emotionally supportive, and positive parenting behavior, and teacher–student relationship quality – mediated the positive association between SES and academic achievement (Baker et al., 2018; Chen, Kong, Gao, & Mo, 2018; Eamon, 2002; Larson et al., 2015; Marks et al., 2006; Xuan et al., 2019). Importantly, with the temporal precedence criterion, only three of six (50%) studies reported significant mediation.

Six studies examined composite measures of stress, support, and stimulation such as material resources and overall parenting practices, as well as overall quality of the home environment, and all (100%) found significant indirect effects. (Chien & Mistry, 2013; Crosnoe & Cooper, 2010; Dupere et al., 2010; Larson et al., 2015; Marks et al., 2006; Mistry, Biesanz, Taylor, Burchinal, & Cox, 2004). Among these, two of four studies (50%) found parent depression to mediate the association of SES with academic achievement (Crosnoe & Cooper, 2010; Mistry et al., 2004). Findings did not change under the temporal precedence criterion.

##### Child characteristics and cognitive/language ability

Several studies (*n* = 39) examined whether children's characteristics, such as their cognitive and language ability acted as mediators of the association between SES and academic achievement. Twenty‐five of 27 studies (93%) studies found that greater general cognitive ability and EF, including effortful control, working memory, and cognitive flexibility, mediated the positive association between SES and academic achievement (Albert et al., 2020; Bachman et al., 2022; Barnes, Boedeker, Cartwright, & Zhang, 2022; Blakey et al., 2020; Cadima, Gamelas, Mcclelland, & Peixoto, 2015; Chevalère et al., 2023; Crook & Evans, 2014; Dilworth‐Bart, 2012; Dolean, Melby‐Lervåg, Tincas, Damsa, & Lervåg, 2019; Ellefson, Zachariou, Ng, Wang, & Hughes, 2020; Fitzpatrick et al., 2013; Galindo & Sonnenschein, 2015; Greenfader, 2019; Korzeniowski, Ison, & Difabio, 2017; Kriegbaum & Spinath, 2016; Lawson & Farah, 2017; Merz et al., 2014; Murphy, Zhang, & Gatzke‐Kopp, 2022; Myrberg & Rosén, 2008; Nesbitt et al., 2013; Perry, Dempster, & McKay, 2017; Rosen et al., 2019; Sun et al., 2018; Waters, Ahmed, Tang, Morrison, & Davis‐Kean, 2021; Zhang, Hu, Ren, & Zhang, 2019). Findings did not change under the temporal precedence criterion (18/20; 90%). Of note, findings for inhibitory control were mixed, with some studies (50%) finding it to be a significant mediator of the association between SES and achievement (Cadima et al., 2015; Greenfader, 2019; Zhang et al., 2019) and others (50%) finding it not to be (Albert et al., 2020; Rosen et al., 2019; Waters et al., 2021).

Children's language abilities also mediated the SES‐achievement association in 12 of 14 (86%) studies (Albert et al., 2020; Cheng & Wu, 2017; Dolean et al., 2019; Fitzpatrick et al., 2013; Forget‐Dubois et al., 2009; Greenfader, 2019; Liu, Chung, & McBride, 2016; Lurie et al., 2021; Schneider, Abel, & Maguire, 2023; Slusser, Ribner, & Shusterman, 2019; Zhang et al., 2013, 2019). Two studies found that externalizing and behavior problems mediated the association between SES and academic achievement (Crosnoe & Cooper, 2010; Eamon, 2002). Findings did not change under the temporal precedence criterion (9/11; 82%).

##### Broader contextual variables

Five studies found school‐level factors (Ainsworth, 2002; Chien & Mistry, 2013; Dupere et al., 2010; Lei, 2018; Marks et al., 2006) and neighborhood resources and activities (Lei, 2018) to mediate the association of SES with academic achievement. Findings did not change under the temporal precedence criterion.

##### Other factors

All 10 studies (100%) that examined educational expectations found that they mediated the association between SES and academic achievement (Ainsworth, 2002; Bodovski & Farkas, 2008; Carolan, 2016; Galindo & Sonnenschein, 2015; Larson et al., 2015; Lei, 2018; Long & Pang, 2016; Ren, Zhang, Jiang, & Huang, 2021; Speybroeck et al., 2012; Yeung et al., 2022). Educational expectations were assessed at the parent, adolescent, neighborhood, and teacher‐level. Importantly, findings did not change under the temporal precedence criterion (5/5; 100%). Similarly, all three studies (100%) that explored academic self‐efficacy, which refers to the students' beliefs and attitudes toward their capabilities to achieve academic success, found that it mediated the association between SES and academic achievement (Chevalère et al., 2023; Kriegbaum & Spinath, 2016; Perry et al., 2017).

Caregiver involvement in school mediated the association between SES and academic performance in three of four studies (75%) (Crosnoe & Cooper, 2010; Galindo & Sonnenschein, 2015; Wolf & McCoy, 2019). Preschool attendance and child‐care quality mediated the association between SES and achievement in three (100%) studies (Dupere et al., 2010; Larson et al., 2015; Sun et al., 2018). Peer contagion and cognitive skills did not mediate the association of SES with academic outcomes in two studies (Coley, Spielvogel, & Kull, 2019; Lei, 2018). Factors investigated in one study each included maternal health, classroom structural quality, birthweight, family structure, mothers age, breastfeeding, student and parent health, student engagement, nutritional environment, and teachability culture (Agirdag, 2018; Barr, 2015; Larson et al., 2015; McCoy, Connors, et al., 2015; Tomaszewski, Xiang, & Western, 2020; Yu, 2007). See Table S5 for details.

#### Moderation

##### Stress, support, and stimulation

One study found that children from lower SES families who experienced stressors, in the form of higher levels of teacher–child conflict, exhibited lower academic achievement (McCormick, O'Connor, & Parham Horn, 2017), which suggests that stress may be a risk factor for lower achievement among low SES youth. Two studies reported that student–teacher closeness benefitted children from low SES households (McCormick et al., 2017; Olsen & Huang, 2021). Measures of stress, stimulation, and support, including higher authoritative parenting, and shared book reading attenuated the association between low SES and lower academic achievement in two studies (Shahaeian et al., 2018; Xia, 2020). Specifically, shared reading at home at home was more strongly associated with early academic skills for children from lower SES and middle SES families compared to higher SES ones (Shahaeian et al., 2018). This suggests that the achievement of children from low SES families may benefit from shared reading activities. In contrast, Galindo & Sonnenschein (2015) found that children from the highest SES quintile benefitted most from learning and reading activities at home relative to the fourth quintile. Low and high SES students saw similar gains in math achievement if they participated in a summer program (both groups made moderately larger mathematics achievement gains than students who did not participate) (Little, Adelson, Kearney, Cash, & O'Brien, 2018). See Figure 3C.

##### Child cognitive and language ability and other characteristics

Two studies found that among children from low SES families, learning motivation (Chen et al., 2018), and a composite measure of learning behaviors (conceptualized as attention/persistence, competence/motivation, and attitude toward learning) (Beisly et al., 2020), was associated with higher achievement, which suggests that learning motivation and positive learning behaviors may benefit the academic achievement of children low SES backgrounds.

##### Other factors

Having a growth mindset buffered the association of poverty with lower academic achievement in one (Claro, Paunesku, & Dweck, 2016) of three studies (33%). One study found that higher body weight exacerbated the negative influence of lower SES on academic achievement (Kranjac & Kranjac, 2021), which suggests that high body weight may be a risk factor for lower academic achievement among low SES youth. Parent involvement in children's education was more positively associated with performance for children whose parents have a school or college education than children whose parents did not attend school (Koepp, Gershoff, & Marteleto, 2022). This suggests that parent involvement in education may not positively influence academic achievement for children whose parents have lower educational achievement. Among children from low SES families or neighborhoods, preK attendance and high preschool SES and quality was associated with higher performance in four of five studies (80%) (Dearing, McCartney, & Taylor, 2009; Laurin et al., 2015; Li et al., 2016; Pearman, 2020), which suggests that preschool attendance and quality may act as a protective factor. Finally, at low and mean levels of household poverty, girls were at higher risk of school dropout (Boyes, Berg, & Cluver, 2017). See Table S6 for details.

## Discussion

The goal of this systematic review was to comprehensively characterize the mediators and moderators of the association of SES with EF, language ability, and academic achievement. To this end, we conducted a systematic synthesis of 136 studies, and clustered variables into commonly examined categories: stress, support, stimulation, child characteristics, and other contextual factors including schools and neighborhoods. Our synthesis of the findings revealed that stress, support, and stimulation, as well as educational expectations, self‐efficacy, classroom and school environment, and teacher–student relationships mediated the association of SES with EF, language ability, and academic achievement, with findings being most consistent for academic achievement as an outcome.

Stimulation – which is the availability of enriching environmental inputs that facilitate learning opportunities for children, typically operationalized as educational resources and learning materials at home and learning and reading activities – was consistently found to mediate the association of SES with EF, language ability, and academic achievement as well as buffer the association of low SES with low academic performance and achievement. These findings lend credence to the notion that cognitive stimulation is a particularly important determinant of children's cognitive development (Rakesh, McLaughlin, et al., 2024), and that interventions targeting cognitive stimulation would be beneficial for children from low SES families. Some argue that the lack of stimulation is merely an ‘epiphenomenon’ of parents with low education or IQ, suggesting that low cognitive stimulation in such households reflects broader issues of parental capacity rather than a direct causal pathway. Further, it is also important to acknowledge genetic confounding. Genetic influences may partly determine both the cognitive stimulation children receive and their inherent cognitive abilities. However, recent research based on a large sample of children indicates that early childhood stimulation has a positive association with intelligence, independent of polygenic scores for IQ (Sánchez‐Luquez et al., 2024). Further bolstering this argument is evidence that cognitive stimulation itself plays a causal role in cognitive development. Indeed, intervention studies designed to increase cognitive stimulation have consistently shown benefits for cognitive outcomes (Korzeniowski et al., 2017; Obradović, Yousafzai, Finch, & Rasheed, 2016; Reina‐Reina, Conesa, & Duñabeitia, 2023). For example, two school‐based stimulation programs in Argentina effectively enhanced EF, leading to improved reading, writing, and math abilities, though the extent of improvement varied with intervention duration and children's ages (Korzeniowski & Ison, 2017). In a 2‐year randomized controlled trial from Jamaica, children with stunted growth received nutritional supplementation and/or cognitive stimulation interventions. By age of 31, those who underwent stimulation, with or without supplementation, showed significantly higher IQ, improved cognitive flexibility, reduced depressive symptoms, and better psychosocial outcomes compared to those without intervention (Walker et al., 2022). These findings suggest that cognitive stimulation plays a causal role in children's development, highlighting the benefits of targeted interventions aimed at enhancing cognitive abilities in disadvantaged populations.

It is also important to note, however, that reciprocal interactions exist between children's cognitive abilities and the stimulation they experience, which add complexity to the issue. Behavioral genetic models reveal that children's initial cognitive abilities predict the level of cognitive stimulation provided by parents (Tucker‐Drob & Harden, 2012). This suggests that environmental inputs ultimately amplify genetic differences (Tucker‐Drob & Harden, 2012). These reciprocal interactions highlight the need for nuanced intervention designs. Children with lower initial cognitive abilities may benefit from structured cognitive stimulation interventions and unstructured environments might necessitate additional support for effective engagement.

Further, our synthesis revealed that support, particularly emotionally supportive parenting behaviors, mediated the association of SES with EF and academic achievement. However, there was less evidence for the mechanistic role of support in the association between SES and language outcomes. A few studies also highlighted its protective role in the association of low SES with low academic achievement. Conversely, while findings were somewhat mixed, exposure to stressors, including parental stress, subjective financial strain, and more parental mental health problems, mediated the association of low SES with lower EF and academic achievement in a few studies. More studies finding cognitive stimulation to be a mediator in links between SES and cognitive and language outcomes is consistent with the notion that deprivation, which is the absence of enriching cognitive and social inputs, rather than other types of adversities (e.g. threat), is associated with cognitive outcomes like EF and language ability (McLaughlin, Sheridan, & Lambert, 2014) – an idea that has been supported using network analyses (Sheridan, Shi, Miller, Sahali, & McLaughlin, 2020). Findings from our review further highlight that support and stress are also pathways through which SES shapes achievement and EF but not language outcomes, the latter of which is shaped primarily through stimulation. These results align with the notion that a secure and positive emotional environment contributes positively to children's cognitive development, whereas environments that induce stress may impede it (Diamond, 2013). This likely occurs because positive and non‐stressful environments allow children to explore and learn from their environments with ease, focus their attention on tasks, and promote positive emotions (Valcan, Davis, & Pino‐Pasternak, 2018), thus enabling them to benefit maximally from the resources available to them. Importantly, support variables found to mediate SES‐achievement links included those beyond the household. Specifically, teacher–student closeness was found to buffer the association between SES and achievement in multiple studies (McCormick et al., 2017; Olsen & Huang, 2021). Indeed, teacher–student relationships play a key role in student engagement and achievement (Hamre & Pianta, 2001). While further work is required to validate these findings, our review highlights that promoting teacher–student relationships for children from low SES backgrounds may be a promising avenue for future research aimed at identifying intervention targets. Further, stress, support, and stimulation mediated links of SES with EF and language ability which in turn mediated the relationship between SES and academic achievement. This suggests a pathway whereby SES influences stress, support, and stimulation, contributing to differences in EF and language, which in turn ultimately influence academic achievement.

In addition, educational expectations and academic self‐efficacy and self‐concept emerged as important mediators in the association of SES with academic achievement. Parents with higher educational expectations may foster a more academically oriented environment at home, instilling greater motivation for learning in their children. This, in turn, can positively impact a child's academic engagement and performance. Importantly, in addition to the parent, children's own academic expectations as well as teacher's educational expectations of the child also mediated the association between SES and achievement. These findings align with recent research on the role of growth mindset in children's achievement (Yeager et al., 2019). For example, a recent study showed that a short online intervention aimed at promoting a growth mindset by emphasizing that intellectual abilities can be developed, contributed to an increase in grades among lower‐achieving students. Additionally, it resulted in increased enrollment in advanced mathematics courses across a nationally representative sample of secondary education students (Yeager et al., 2019). Other work has shown that the belief that SES can be changed leads to greater student engagement and academic achievement (Zhao et al., 2021). These findings underscore the importance of considering educational expectations (at student, parent, and teacher‐level) in promoting resilience among youth from low SES backgrounds. Further research is needed to confirm these findings across cultural contexts. Exploring the effectiveness of interventions targeting educational expectations in low SES families and schools in disadvantaged neighborhoods, such as workshops or counseling programs to raise the educational expectations of parents, children, and teachers, without imposing undue academic pressure on youth, may offer a promising direction for future research in bridging the achievement gap.

### Designing effective interventions

#### Reducing poverty

Most importantly, while understanding the mechanisms through which poverty influences children's cognitive abilities and academic achievement is crucial for informing targeted interventions, it cannot be stressed enough that the fundamental solution lies in reducing poverty itself. The high prevalence of poverty during childhood (ALICE in Focus, 2022; The Annie E. Casey Foundation, 2019) and its profound influence on children's development underscore the necessity of structural changes to mitigate these effects. The United Nations Sustainable Development Goal (SDG) 10 calls for the reduction of ‘inequality within and among countries’, emphasizing that economic policy changes promoting equitable income distribution, such as universal basic income schemes, are vital for reducing inequality and poverty (Piketty & Goldhammer, 2014). Of note, however, the origins of poverty are complex and reducing it will require both structural changes (e.g. economic reform) as well as individual level interventions. For example, interventions that provide access to affordable and effective mental health care for parents can help address issues of depression, anxiety, substance use, and other mental health conditions that can impact parents' ability to participate in the labor force. Together, such changes will ultimately contribute to positive child developmental outcomes. Indeed, recent work shows that increases in income can enhance cognitive stimulation at home and improve child cognitive outcomes. For example, the Baby's First Years study, which provides cash transfers to low‐income families, shows that higher cash gifts lead to higher spending on educational resources and more time spent on activities like reading with children (Gennetian et al., 2022). Therefore, financial support not only reduces stress for parents but also boosts the quality of parenting interactions, encouraging positive and stimulating behaviors that benefit children's cognitive development.

#### Provision of high‐quality child care

Our findings highlight the importance of early childhood education. Studies show that children from lower‐income families benefit more from high‐quality child care (Peisner‐Feinberg, 2007), which can shield them from the adverse effects of living in disadvantaged environments and compensate for economic disadvantages by preparing children for school (Canadian Council on Learning, 2006). Indeed, UNESCO has emphasized the pressing need to improve access and quality of early child care to achieve the United Nations SDG 4, which aims for inclusive and quality education for all (UNESCO, 2024). Structured, high‐quality child care promotes essential cognitive and social skills, and since non‐profit child‐care centers tend to provide higher quality care than for‐profit ones (Canadian Council on Learning, 2006; Cleveland, 2023), the onus falls on governments to facilitate the availability of such centers. Countries like Sweden and France offer universal child care, ensuring all children have access regardless of their parents' income – a model that should be adopted by other countries. Increasing access to high‐quality child care will also allow parents to participate in the labor force, thus contributing to the mitigation of poverty. From an economic perspective, the benefits of quality child care, such as longer school attendance, higher future earnings, and reduced crime rates, justify government regulation and financial support for child‐care services (Karoly & Bigelow, 2005).

#### Targeting cognitive stimulation

Given the evidence that cognitive stimulation positively influences EF, language ability, and academic achievement, interventions should focus on enhancing stimulation for children from low SES backgrounds. Research shows that toddlers' cognitive skills are more strongly influenced by availability of learning materials, parental reading, involvement in activities, and quality language input than by parents' socioeconomic status or education level (Malhi, Menon, Bharti, & Sidhu, 2018). As such, interventions should focus both on providing stimulating learning materials, as well as the relational aspect of cognitive stimulation and parent–child interactions (Rakesh, McLaughlin, et al., 2024). Training programs that help parents learn to balance guidance with fostering independence, and programs that leverage applications that facilitate shared reading and learning activities (e.g. TANDEM; Early Ideas Limited, 2023), could be effective in narrowing achievement gaps.

Beyond the home, school‐based programs could be designed to integrate the abovementioned high‐quality learning resources into daily activities, ensuring that all children, particularly those from disadvantaged backgrounds, have regular access to stimulating educational experiences. Furthermore, partnerships with local libraries and community centers to offer additional learning opportunities and workshops can reinforce the cognitive stimulation provided at home and in schools. Finally, interventions should be adaptable to the cognitive abilities of children. For example, as we stated earlier, children with lower initial cognitive abilities might benefit from more intensive and structured cognitive stimulation programs, whereas those with higher cognitive abilities could be offered enrichment activities that challenge and expand their existing skills. Tailoring interventions to meet these needs can help maximize the effectiveness of cognitive stimulation efforts.

#### Promoting emotional support

Numerous interventions have been shown to increase parental support and foster improvements in the parent–child relationship (e.g. Kehoe, Havighurst, & Harley, 2020; Zhang, Wang, & Li, 2024). Recently developed applications like ‘Parenting Matters’ that teach parents both love and limits can be leveraged for such interventions (Parenting Matters, 2024). In educational settings, enhancing teacher‐student relationships through professional development programs can improve teacher practices and increase student engagement, particularly for those from low SES backgrounds. Initiatives could include mentorship programs where teachers receive training in building strong, supportive relationships with their students.

#### Fostering educational expectations and self‐efficacy

Educational expectations and self‐efficacy are important factors influencing academic achievement. Parents' expectations have previously been shown to predict changes in achievement. That is, they influence future achievement over and above the effect of prior achievement (Pinquart & Ebeling, 2020). Interventions could therefore aim to cultivate high educational expectations among parents, children, and teachers without putting undue pressure on students. Studies have shown that raising teacher expectations is both achievable can boost student achievement (de Boer, Timmermans, & van der Werf, 2018). Schools should therefore implement programs that engage parents and teachers in setting high academic goals for children and provide them with tools to support these goals.

### Future directions

#### Focus on the factors at the level of the household as mediators and moderators

Most studies included in the present review investigated stress, support, and stimulation that occurs within the context of the household, particularly the parent child relationship. However, they occur in varied contexts. For example, as mentioned above, the home environment has been shown to be the most important, but not the only setting for cognitive stimulation (Crosnoe, Leventhal, Wirth, Pierce, & Pianta, 2010), and stress, support, and stimulation can also occur in the context of relationships with peers, siblings, grandparents, extended family, teachers, and even neighbors, to name a few. Further, children live in complex environments that operate at micro‐, meso‐, and macro levels (Bronfenbrenner, 1977). For example, neighborhood environments play a crucial role in child development (Leventhal & Brooks‐Gunn, 2000) and children cumulatively spend over 15,000 hr at school (Rutter, Maughan, Mortimore, Ouston, & Smith, 1979), yet these factors were only examined in a few studies. While not widely investigated, several studies also highlighted the important role of broader contextual factors, such as neighborhood resources and availability of extracurricular activities, and school environment, including classroom environment and quality and teaching practices and stress, in the association of SES, particularly at the neighborhood or school level, and EF, language ability, and academic achievement. Several factors beyond the parent–child dyad that are likely important for child developmental outcomes were not investigated in studies. These included relationships with peers, bullying and victimization, screentime habits, access to quality healthcare, nutrition, and mental health services and support, immigration status and acculturation experiences, substance use behaviors, discrimination and systemic barriers, sleep quality, noise, residential density, collective efficacy, and access to greenspaces, parks, and libraries. These factors likely intersect with SES in meaningful ways to predict children's outcomes and warrant further investigation. Future work should look beyond household level factors to gain a more comprehensive understanding of the role of different factors in association between SES and children's cognitive and academic outcomes. Such work would help identify additional areas for intervention and support.

#### Understanding the role of the setting and medium of cognitive stimulation

While prior work has shown that the home environment is an important, but not the only, setting in which stimulation is important for early learning (Crosnoe et al., 2010), most work reviewed in the present study investigated the role of stimulation in the home environment. As a result, the question of how important settings outside the home are for stimulation, particularly in low SES contexts, remains unanswered (Rakesh, McLaughlin, et al., 2024). Further, beyond the setting, the medium of delivery of cognitive stimulation is also important to consider. Digital experiences also have the capacity to shape children's cognitive development. However, only one study included in the present review examined the role of computer resources as a mediator in the link between SES and EF, and reported a significant indirect effect (Lipina et al., 2013). Affordable tablets and smartphones can provide access to educational apps, online learning platforms, and interactive games, even in resource‐scarce settings. A study conducted in Malawi demonstrated that a cognitive stimulation intervention delivered through digital means improved mathematics performance in low‐income settings (Pitchford, 2014). Similarly, an intervention from Indonesia found that iPad‐based tasks designed to promote cognitive abilities along with nutritional supplementation in the form of fortified milk powder resulted in a significant increase in cognitive performance and a greater reduction in attentional problems (Schneider et al., 2018). Additional advantages of digital tools include the fact that they can be adaptive and therefore tailor content and activities to individual learning paces and needs, providing a personalized learning experience. Nonetheless, a recent pilot randomized trial of a child development app indicates that mobile health interventions might not be effective on their own in facilitating behavior change among low‐income caregivers due to limited participation (Cunningham et al., 2023). This highlights that if digital interventions are created, parents must be trained to use them.

However, as described earlier, few studies included in the present review examined whether the setting (e.g. child‐care centers, home, school) and method (e.g. availability of materials, parental scaffolding, self‐guided or parent‐guided use of digital learning applications) of delivering cognitive stimulation play a role in the association of SES with outcomes, warranting further investigation. Further, stimulation in different settings can also interact with one another. For example, Cabrera, Jeong Moon, Fagan, West, and Aldoney (2020) show that children derive greater advantages from their child‐care experience when they experience higher levels of cognitive stimulation at home. Hence, it is crucial for forthcoming research not only to explore the role of stimulation across diverse environments in cognitive and academic outcomes in low SES contexts but also to investigate the potential interactions between stimulation in these settings. Such research is crucial to help determine maximally effective intervention approaches.

#### Methodological variation, consideration of other SES indicators, and neurocognitive domains

Given the considerable heterogeneity in the measurement of key variables of interest – namely, SES, the mediators and moderators, EF, language, and achievement – as well as statistical approaches, a meta‐analysis was not feasible. In particular, few of the mediators and moderators were measured using similar approaches across studies. For example, the way cognitive stimulation was operationalized differed from study to study, as did the operationalization of other variables. This makes it challenging to draw firm conclusions regarding the more specific aspects of stress, support, and stimulation that explain links between SES and academic achievement. With regard to SES, it was most frequently operationalized as parent education, income (or income adjusted for household size), and composite indices that capture various dimensions of household SES. In contrast, area‐level SES and school SES were utilized less frequently despite the important role of neighborhood and school environments for child development (Leventhal & Brooks‐Gunn, 2000; Raniti, Rakesh, Patton, & Sawyer, 2022). Neighborhood and school SES may act through both similar and distinct mechanisms than household SES. For example, lower neighborhood SES is associated with greater exposure to violence in the neighborhood and exposure to toxins but also lower utilization of education‐focused practices (Greenman, Bodovski, & Reed, 2011) and several parenting behaviors such as parental warmth and monitoring (Burton & Robin, 2000; Klebanov, Brooks‐Gunn, & Duncan, 1994; Shumow & Lomax, 2009). Yet, few studies have considered both household and area‐level SES and their putative mechanisms – an important direction for future work. In addition, wealth, a less commonly used indicator of SES, is distributed more unequally than income (Desilver, 2015). There has been a decrease in median net worth, while mean net worth has increased disproportionately over the last three decades (Pfeffer & Schoeni, 2016). Indeed, wealth has been shown to predict children's outcomes over and above other indicators of household SES (Miller, Podvysotska, Betancur, & Votruba‐Drzal, 2021), yet only a few studies have considered the environmental mechanisms through which wealth may influence children's cognitive, language, and academic outcomes – an important avenue for future research to consider.

#### Research from low‐ and middle‐income countries

Most studies included in the present review were conducted using data from high‐income countries, a known issue in developmental research (Draper et al., 2023; Nketia, Amso, & Brito, 2021). Since only a limited number of studies included in this review were conducted in low and middle countries, we are unable to make inferences about whether the observed mechanisms of the association between SES and cognitive and academic outcomes are the same in different cultural contexts. Such research is needed to inform targeted interventions.

#### Understanding the underlying biological mechanisms

Progress in neuroscience that shows that brain development is nonlinear and goes well into young adulthood (Norbom et al., 2021) has revealed fresh possibilities for implementing effective interventions in older children, extending beyond traditionally defined critical periods (Nelson, Sullivan, & Engelstad, 2024). Indeed, childhood and adolescence are characterized by rapid brain development (Norbom et al., 2021; Rakesh, Dehestani, & Whittle, 2024). Longitudinal studies indicate that the rapid development of children's brains during this period makes them highly responsive to both positive and negative environmental influences that can impact their development (Farber, Gee, & Hariri, 2020; Hanson et al., 2013; McLaughlin, Weissman, & Bitrán, 2019; Pozzi et al., 2023; Rakesh et al., 2021; Rakesh, Allen, & Whittle, 2021; Rakesh, Elzeiny, Vijayakumar, & Whittle, 2023; Rakesh, Whittle, Sheridan, & McLaughlin, 2023; Rakesh, Zalesky, & Whittle, 2022; Whittle et al., 2014, 2022; Whittle, Zhang, & Rakesh, 2024). Factors such as parenting and school environments may interact with SES in predicting brain structure and function, which can in turn influence cognitive and academic outcomes (Brody et al., 2019; Rakesh et al., 2021; Rakesh, Seguin, Zalesky, Cropley, & Whittle, 2021; Rosen, Sheridan, Sambrook, Meltzoff, & McLaughlin, 2018; Whittle et al., 2017). Evidence shows that other biological factors, such as inflammation, may also moderate links between SES and EF (Kokosi, Flouri, & Midouhas, 2021). However, few studies to date have examined the neural and biological mediators and moderators of the association between SES, stress, support, stimulation, and children's cognitive, language, and academic outcomes. In particular, there is a need for research that examines changes in brain structure and function pre and post intervention in low SES children and adolescents and how these changes relate to cognitive, language, and academic outcomes (Blair & Raver, 2016). Research in this area is in its nascent stages, but as it progresses, it will provide insight into how SES and its proximal mediators get under the skin to influence child cognitive and academic outcomes.

### Limitations

Our review concentrated on EF, language, and academic achievement due to their robust associations with SES. However, the links between SES and other neurocognitive domains, such as memory, processing speed, attention, and visuospatial skills, might be mediated by factors not addressed in our review. Future studies should delve into these to gain a comprehensive understanding of the mediators and moderators of associations between SES and other cognitive functions. In addition, our review focused on behavioral measures of EF. Behavioral measures and ratings of EF and are not highly correlated they have been suggested to assess different constructs (Toplak et al., 2013). Further, Biederman et al. (2008) demonstrated that impairments identified through performance‐based measures of EF do not necessarily align with impairments observed in ratings of EF, and vice versa. As such, future reviews on the subject may benefit from focusing on parent and teacher‐reported ratings of EF, links with which may be mediated by factors distinct from those identified in our study. Additionally, our review categorized factors into stress, support, and stimulation across various contexts, as well as other contextual factors (e.g. neighborhood and school variables) and individual characteristics, based on their prominent roles in the literature. While this grouping is theoretically sound, it may have influenced the findings, and an alternative categorization could potentially yield different conclusions.

### Conclusion

Taken together, these findings fill an important gap in the evidence base by systematically characterizing the proximal mechanisms and factors that may buffer the association of SES with children's EF, language, and academic outcomes. Findings show the important role that stress, support, stimulation, as well as neighborhood‐ and school‐level factors play in these associations. Interventions should focus on reducing poverty, improving access to early child care, promoting positive parenting and teacher behaviors, providing cognitive stimulation, and fostering high educational expectations. More work that looks beyond the household, adapts to the changing landscape of technology, and aims to understand heterogeneity and uncover neural and biological mechanisms may spark further advances in our understanding of the pathways that link SES with cognitive and academic outcomes and point to new avenues for early interventions to reduce socioeconomic disparities.

**Key points**

**What's known?**

- Low socioeconomic status is associated with lower cognitive performance and academic achievement. Despite extensive research documenting SES‐related differences in these domains, our understanding of the mechanisms underlying these associations and factors that may mitigate these relationships is limited.

**What's new?**

- We conducted a systematic review of 136 studies to identify the mediators and moderators in the association of SES with EF, language ability, and academic achievement. Stress, support, stimulation, and broader contextual factors at the school- and neighborhood level are mediators and protective factors in these associations.

**What's relevant?**

- These findings have relevance for intervention studies aimed at mitigating socioeconomic disparities in children’s achievement.

## Supporting information


**Table S1.** Mediation findings for EF.
**Table S2.** Moderation findings for EF.
**Table S3.** Mediation findings for language ability.
**Table S4.** Moderation findings for language ability.
**Table S5.** Mediation findings for academic achievement.
**Table S6.** Moderation findings for academic achievement.
Search terms.
Quality assessment rating.
