# Supplementary material for: Annual Research Review: Associations of socioeconomic status with cognitive function, language ability, and academic achievement in youth: a systematic review of mechanisms and protective factors
Source: J Child Psychol Psychiatry. 2024 Dec 3;66(4):417–39. doi: 10.1111/jcpp.14082 (PMC11920614; doi:10.1111/jcpp.14082)
Supplement: Supplementary file 1 — Table S1. Mediation findings for EF. Table S2. Moderation findings for EF. Table S3. Mediation findings for language ability. Table S4. Moderation findings for language ability. Table S5. Mediation findings for academic achievement. Table S6. Moderation findings for academic achievement. Search terms. Quality assessment rating. [file JCPP-66-417-s001.pdf]

Supplementary Material for "Associations of socioeconomic status with cognitive function, language ability, and academic achievement in youth – a systematic review of mechanisms and protective factors"

Table of Contents

Table S1. Mediation findings for EF 2
Table S2. Moderation findings for EF 6
Table S3. Mediation findings for language ability 8
Table S4. Moderation findings for language ability 14
Table S5. Mediation findings for academic achievement 15
Table S6. Moderation findings for academic achievement 28
Search terms 32
Quality assessment rating 32

Table S1. Mediation findings for EF

AUTHOR, DATE	SAMPLE INFORMATION (N, AGE RANGE/MEAN AGE + SD FOR EF MEASUREMENT)	SES MEASURE	MEDIATOR	OUTCOME	MAIN FINDINGS	KEY DIMENSION
**BAKER ET AL. (2020)**	106, 52.8 ± 6.6 months	Household income	Subjective financial strain	Hot (i.e., emotionally salient) and cool (i.e., not emotionally salient) EF	Higher household income was associated with less parental subjective financial strain, which in turn mediated the positive association between household income and cool EF performance.	Stress
**DANERI ET AL. (2018)**	1,009, T1 = 6 months, T2= 15 months, T3= 24 months, T4 = 36 months, T5= 48 months	Composite SES	Maternal number of different words (15 months, 24 months, 36 months), maternal language complexity (15 months, 24 months, 36 months)	Composite EF (at 48 months)	Low SES was associated with lower maternal number of different words at 24 months and lower maternal language complexity at 36 months, which mediated the association of low SES with lower EF.	Stimulation
**DILWORTH-BART ET AL. (2009)**	73, 24-months	Composite SES	Maternal emotion scaffolding, maternal attention scaffolding	Verbal working memory, non-verbal working memory	Low SES was associated with less maternal emotional and attentional scaffolding, which in turn mediated the association between low SES and lower verbal working memory.	Support, Stimulation
**ENCINGER ET AL. (2020)**	249, 53.98 ± 6.46 months	Marginal food security	Parental stress	Self-regulation	Increased parenting stress mediated the association between marginal food security and lower self-regulation.	Stress
**HACKMAN ET AL. (2015)**	1,009, (longitudinal sample, with outcomes at 54 months and Grades 1, 3, and 5 depending on the specific measure; mediators were measured earlier in development). Mediation analyses focused on model intercepts (i.e., at 54 months).	Income to needs ratio, maternal education	Infant/toddler home enrichment, early childhood home enrichment, infant/toddler maternal sensitivity, early childhood maternal sensitivity, stressful life events experienced by the family, birthweight, gestational age, postpartum depression, parent stress in infancy	Working memory, planning abilities	High income to needs ratio was associated with higher infant/toddler and early childhood home enrichment and infant/toddler and early childhood maternal sensitivity. These variables mediated the positive association between income to needs ratio and working memory. Higher income to needs ratio and higher maternal education were associated with greater early childhood home enrichment and maternal sensitivity. These variables mediated the positive association of income and education with planning abilities.	Stimulation, Support

AUTHOR, DATE	SAMPLE INFORMATION (N, AGE RANGE/MEAN AGE + SD FOR EF MEASUREMENT)	SES MEASURE	MEDIATOR	OUTCOME	MAIN FINDINGS	KEY DIMENSION
**HE & YIN (2016)**	157 families, low-SES family children: 83, 9.93 years ± 1.27; middle-SES family children, 74, 10.00 years ± 1.35	Composite SES	Negative affect, positive affect, self-reported stress	Cognitive flexibility, inhibitory control, working memory, planning	Low SES was associated with increased negative affect and increased self-reported stress which in turn mediated the association between low SES and lower EF.	Stress, CC
**LIPINA ET AL. (2013)**	250, 4.87 years ± 0.59	Composite SES	Literacy activities, computer resources	Fluid processing, working memory, attention control, planning	High SES was associated with higher literacy activities which in turn mediated the positive association of SES with fluid processing and working memory. High SES was associated with higher computer resources, which mediated the positive association between SES and fluid processing and attention control.	Stimulation
**MCCOY, ZUILKOWSKI ET AL. (2015)**	2,711, 6.21-years-old, 5 - 7 years	Wealth, caregiver education	Home-based cognitive stimulation, years of early childhood education, physical development (i.e., height for age)	Composite EF	Wealth and education were positively associated with cognitive stimulation, which in turn mediated the positive association of wealth and education with EF. In addition, wealth was positively associated with years of early childhood education, which mediated the association between wealth and EF. In the multiple mediation models including physical development, wealth was positively associated with physical development via years of early childhood education. This path mediated the association of wealth with EF.	Stimulation, Other
**ROSEN ET AL. (2019)**	101, 5.55 years ± 0.37, 60-75 months	Income to needs ratio, parent education	Cognitive stimulation, language exposure (quality and quantity)	Working memory, inhibition, cognitive flexibility	Income to needs ratio and higher parent education were positively associated with cognitive stimulation, which mediated the positive association between SES and working memory, inhibition, and cognitive flexibility. Parent education was positively associated with language exposure which in turn mediated the positive association between parent education and inhibition.	Stimulation
**SARSOUR ET AL. (2011)**	60, 9.9 years ± 0.96, 8-12 years	Composite SES	Home environment composite, physical environment, enrichment activities, parental responsivity, encouragement of maturity, emotional climate/acceptance, learning materials and opportunities, family	Working memory, cognitive flexibility, inhibitory control	High SES was positively associated with overall quality of the home environment, measures of parental responsivity, and family companionship. These variables mediated the positive association between SES and inhibitory control. High SES was positively associated with enrichment activities and family companionship, which in turn mediated the positive association between SES and working memory.	Stimulation, Support, SSS

AUTHOR, DATE	SAMPLE INFORMATION (N, AGE RANGE/MEAN AGE + SD FOR EF MEASUREMENT)	SES MEASURE	MEDIATOR	OUTCOME	MAIN FINDINGS	KEY DIMENSION
			companionship, family integration			
**SUOR ET AL. (2016)**	185 mother-child dyads, T1 = 3.5 years, T2 = 5 years	Composite SES	Maternal harsh discipline, maternal responsiveness	Working memory	Higher SES was associated with less maternal harsh discipline, which in turn mediated the positive association between SES and working memory. In addition, maternal working memory buffered the association between low SES and higher levels of harsh discipline in a moderated mediation model.	Stress
**VOGEL ET AL. (2021)**	1,292 families, ~48 months	Composite SES	Deprivation, threat	Composite EF	Low SES was associated with higher levels of deprivation which in turn mediated the association between low SES and lower EF.	SSS
**VRANTSIDIS ET AL. (2020)**	151, ~36 months	Income to needs ratio, parent education	Maternal psychological distress, harsh parenting, cognitive stimulation	Working memory, inhibitory control, self-control	Low parent education was associated with higher maternal psychological distress which in turn mediated the association between low parent education and lower working memory, inhibitory control, and self-control. Education also had an indirect influence on self-control in the opposite direction through maternal psychological distress and harsh parenting. Increased maternal psychological distress correlated with more harsh parenting practices, which in turn predicted enhanced self-control.	SSS
**WEI ET AL. (2021)**	955 children, 251 teachers in 159 preschools, 4.19 years ± 0.46	Neighborhood SES, neighborhood resources (non-economic)	Preschool classroom quality: teacher emotional support, instructional support, classroom organization	Inhibitory control	NS	
**WOLF & MCCOY (2017)**	2,137 children, 5.16 years ± 1.34	Household wealth, caregiver education	At-home stimulation, caregiver school involvement, no. of books in the household	Composite EF	NS	

AUTHOR, DATE	SAMPLE INFORMATION (N, AGE RANGE/MEAN AGE + SD FOR EF MEASUREMENT)	SES MEASURE	MEDIATOR	OUTCOME	MAIN FINDINGS	KEY DIMENSION
**YU ET AL. (2020)**	359, T1 = 2.5 years, T2 = 3.5 years, T3 = 6 years, T4 = 7 years. Latino and African American; all below the poverty line.	Frequency of exposure to severe poverty	Parental sensitive support, parental intrusiveness	Behavioral self-regulation	Greater exposure to severe poverty was associated with lower levels of maternal sensitive support which in turn mediated the association between exposure to severe poverty and lower growth in behavioral self-regulation (only in Latino children).	Support
**ZHAO ET AL. (2023)**	260, 13.35 years ± 0.65, 12–15 years	Composite SES	Mother behavioral participation, mother intellectual involvement, mother emotional involvement, father behavioral participation, father intellectual involvement, father emotional involvement	Working memory	High SES was associated with higher mother behavioral involvement which in turn mediated the positive association between SES and working memory. High SES was associated with higher mother intellectual involvement which in turn was associated with lower working memory, thus acting as a suppressor in the association of SES with working memory.	Other

Table S2. Moderation findings for EF

AUTHOR, DATE	SAMPLE INFORMATION (N, AGE RANGE/MEAN AGE + SD)	SES MEASURE	MODERATOR	OUTCOME	MAIN FINDINGS	KEY DIMENSION
**BAKER ET AL. (2020)**	106, 52.8 ± 6.6 months, 37 - 64 months	Household income	Parental financial strain	Hot (i.e., emotionally salient) EF performance	There was more variation in the effect of parents' financial strain levels on children's hot EF performance if the children were from relatively lower income families. Low financial strain buffered the association of low income with lower hot EF performance.	Stress
**CUBIDES-MATEUS ET AL. (2022)**	765, T1=52.63 months ± 3.54, 3 time points at fall, winter, and spring of each preschool year	Composite SES	Neighborhood resources	Growth in EF	NS	
**HARTANTO ET AL. (2018)**	11,288-8103, across 4 time points from kindergarten to first grade	Composite SES	Bilingualism	Inhibitory control, verbal working memory, attentional focusing/shifting behaviors	The association between SES and EF across all three domains was less steep in bilinguals (bilingualism buffered the association of low SES with lower EF).	CC
**KAO ET AL. (2018)**	117, 4.18 years ± 0.29	Household income, education, occupational prestige	Parent EF	Composite EF	The positive association between parent EF and child EF was present only in low-income households.	Other
**MING ET AL. (2021)**	885, 10.68 ± 1.07	Composite SES	Subjective SES, subjective social mobility	Inhibitory control, working memory, cognitive flexibility	SES was more positively associated with cognitive flexibility among children whose parents reported low subjective SES and low subjective social mobility but not high subjective SES and high subjective social mobility.	Stress, other
**NEUENSCHWANDER ET AL. (2017)**	171, 68.9 months ± 4.2	School level poverty	Teacher stress	Composite EF	School-level poverty moderated the association between teacher stress and child EF such that when teachers reported high levels of stress, child EF did not differ between high- and low-poverty schools. However, when teachers reported low levels of stress, school poverty seemed to matter; low teacher stress was beneficial to child EF in low-poverty schools, but the opposite was true in high-poverty schools.	Other
**PHILBROOK ET AL. 2017**	282, T1=9.44 ± 0.71, T2=10.37 ± 0.68), T3=11.33 ± 0.69)	Income to needs	Sleep efficiency	Working memory (intercept and change over time)	NS	

AUTHOR, DATE	SAMPLE INFORMATION (N, AGE RANGE/MEAN AGE + SD)	SES MEASURE	MODERATOR	OUTCOME	MAIN FINDINGS	KEY DIMENSION
**PICCOLO ET AL. (2018)**	108, 14.10 years, 9 – 18 years	Family income, parent education	School climate (academic support), school climate (academic support)	Composite EF	Low family income was associated with lower EF performance, but the strength of this association decreased with greater academic support.	BCF
**RAVER ET AL. (2013)**	1,292 children, data collected from low-income families at T1 = 7 months, T2 = 15 months, T3 = 24 months, T4 = 36 months, T5 = 48 months	Chronic exposure to poverty, chronic financial strain, chronic housing risk (assessed using data from multiple time points)	Temperamental reactivity, demographic category membership in race/region (baseline)	Composite EF (48 months)	Chronic exposure to poverty and financial strain were less negatively associated with EF performance at lower levels of temperamental reactivity in infancy.	CC
**RIBNER ET AL. (2017)**	807, 68.9 months ± 4.0	Income-to-needs ratio	Television viewing	Composite EF	High levels of television viewing had a negative association with EF at only lower levels of income to needs ratio.	Other
**ROCHETTE & BERNIER (2014)**	114 mother-child dyads, 36.82 months ± 0.84	Composite SES	Maternal response to positive signals, maternal response to distress, maternal Positive affect sharing, maternal hostility/ rejection, maternal sensitivity/ responsiveness, maternal physical proximity	Conflict EF, impulse control EF	Low SES was associated with lower conflict EF but the strength of this association decreased at higher levels of maternal response to distress. In addition, higher levels of maternal response to both distress and positive signals, and higher maternal physical proximity, buffered the association of low SES with lower impulse control EF.	Support
**SARSOUR ET AL. (2011)**	60, 9.9 years ± 0.96	Composite SES	Single-parent status	Working memory, cognitive flexibility, inhibitory control	The association between SES and inhibitory control and cognitive flexibility was more positive for single parent households than two-parent households. Children from single parent families performed less well relative to children from two-parent families of similar SES.	SSS
**ST. JOHN & TARULLO (2019)**	121, 4.5-5.5 years	Composite SES	Parental well-being, household chaos, neighborhood chaos (measured as neighborhood quality)	Working memory, inhibitory control	SES was more positively associated with inhibitory control and working memory at higher levels of neighborhood 'chaos'.	BCF
**WEI ET AL. (2021)**	955, 251 teachers in 159 preschools, 4.19 years ± 0.46	Neighborhood SES	Neighborhood resources	Inhibitory control	Children demonstrated the highest EF in lower SES but relatively well-resourced contexts.	BCF

Table S3. Mediation findings for language ability

AUTHOR, DATE	SAMPLE INFORMATION (N, AGE RANGE/MEAN AGE + SD FOR LANGUAGE ABILITY MEASUREMENT)	SES MEASURE	MEDIATOR	OUTCOME	MAIN FINDINGS	KEY DIMENSION
**BAYDAR & AKCINAR (2015)**	902, 36 - 47 months	Maternal education, composite economic status (based on household goods, physical environment, material possessions, expenditures)	Learning materials, stimulation for learning, maternal responsiveness, power assertive parenting, support from the neighbors, physical resources of the neighborhood	Receptive vocabulary	Higher levels of learning materials and stimulation for learning mediated the positive association of maternal education and economic status with language ability. In addition, maternal responsiveness mediated the association of economic status with language ability.	Stimulation, Support
**BETANCOURT ET AL. (2015)**	54, ~7 months	Composite SES	Maternal vocabulary	Composite language ability, expressive communication, auditory comprehension	NS	
**CHEUNG & WONG (2020)**	139, T1=final year of kindergarten and T2=second year of primary school	Household income	Parental education investment, parental stress, parent authoritativeness	Receptive vocabulary	Low income was associated with lower parental education investment which mediated the association between low income and lower receptive vocabulary at T2 (via receptive vocabulary at T1). Low income was associated with higher parental stress which was associated with lower parental authoritativeness which in turn mediated the association between low income and lower receptive	Stimulation, SSS

AUTHOR, DATE	SAMPLE INFORMATION (N, AGE RANGE/MEAN AGE + SD FOR LANGUAGE ABILITY MEASUREMENT)	SES MEASURE	MEDIATOR	OUTCOME	MAIN FINDINGS	KEY DIMENSION
					vocabulary at T2 (via receptive vocabulary at T1).	
**CODDINGTON ET AL. (2014)**	1,589, 50.16 months, 48-58 months	Maternal education, household income	Standard of living, home ownership, cognitive stimulation, linguistic stimulation, publicly funded center-based ECCE, privately funded center-based ECCE	Vocabulary	Higher maternal education was associated with higher standard of living, cognitive stimulation, and linguistic stimulation. These variables mediated the positive association of maternal education with child vocabulary. High income was associated with higher standard of living which in turn mediated the positive association between household income and child vocabulary.	Stimulation, SSS
**COLEY ET AL. (2019)**	3,396, data from two longitudinal studies between preschool and kindergarten years	Classroom economic composition	Peer cognitive skills, instructional quality	Receptive language	Lower SES, as measured by classroom economic composition, was associated with lower receptive language. This association was mediated by lower peer cognitive skills in the fall and lower instructional quality in the spring.	Other
**DULAY ET AL. (2018)**	667, outcome measured separately for 3-year-olds, 4-year-olds, and 5-year-olds	Composite SES	Preschool attendance, home literacy activities, home literacy environment, parent self-efficacy	Vocabulary	For 3- and 4-year-old children, higher SES was associated with higher preschool attendance which in turn mediated the positive association between SES and vocabulary.	Other
**DUPÉRÉ ET AL. (2010)**	1,364, grade 1	Neighborhood advantage	Home environment quality, childcare environment quality, classroom environment, school advantage, maternal depression	Vocabulary	Higher neighborhood advantage was associated with higher quality home and childcare environment which in turn mediated the positive association between neighborhood advantage and vocabulary.	SSS, Other
**FEKONJA-PEKLAJ ET AL. (2015)**	99, 37.8 months ± 18.3 months	Parent education, index of family possessions	Literacy activities, parents mental transformation during shared play, shared reading	Language ability	NS	
**FOSTER ET AL. (2005)**	325, 59.1 months ± 5.7	Composite SES	Home learning environment, social risk (i.e., the amount of social support available to the primary caregiver)	Emergent literacy (receptive vocabulary, phonemic awareness)	SES was positively associated with home learning environment which in turn mediated the positive association between SES and emergent literacy.	Stimulation

AUTHOR, DATE	SAMPLE INFORMATION (N, AGE RANGE/MEAN AGE + SD FOR LANGUAGE ABILITY MEASUREMENT)	SES MEASURE	MEDIATOR	OUTCOME	MAIN FINDINGS	KEY DIMENSION
**GONZALEZ ET AL. (2016)**	252, 4.71 years ± 0.30	Maternal education, family income	Parent reading belief, home learning environment reading frequency	Expressive vocabulary, receptive vocabulary	The positive association of family income and material education with receptive vocabulary was mediated by higher levels of parent reading belief, home learning environment, book availability and reading frequency (in that order) in a serial mediation model.	Stimulation
**HOFF (2003)**	63, 16-31 months. high-SES mother-child dyads, 20.8 months ± 3.1. mid-SES mother-child dyads, 21.6 months ± 3.0	Composite SES	Maternal speech	Child vocabulary	High SES was associated with higher quality of maternal speech which in turn mediated the positive association between SES and child vocabulary.	Stimulation
**IRUKA ET AL. (2014)**	9,550, 52.95 months ± 4.18	Family income, maternal education	Availability of learning materials, language stimulation, outside activities	Receptive language, expressive language	Family income and maternal education were positively associated with greater availability of learning materials, language stimulation, and number of outside activities, which mediated the positive association of family income and maternal education with receptive and expressive language (findings differed between races: Asians, African Americans, Euro-Americans and Hispanics). In general, language stimulation and outside activities were the most consistent mediators for Euro-Americans, learning materials was the most consistent mediator for African Americans, learning materials and language stimulation were the most consistent mediators for Hispanics, and learning materials and outside activities were the most consistent mediators for Asians.	Stimulation

AUTHOR, DATE	SAMPLE INFORMATION (N, AGE RANGE/MEAN AGE + SD FOR LANGUAGE ABILITY MEASUREMENT)	SES MEASURE	MEDIATOR	OUTCOME	MAIN FINDINGS	KEY DIMENSION
**KOHEN ET AL. (2008)**	3,528, 5.05 years ± 0.86	Neighborhood structural disadvantage	Neighborhood cohesion, family functioning, consistent parenting, depression, punitive parenting, literacy	Verbal ability	The association between neighborhood structural disadvantage and verbal ability was mediated by neighborhood cohesion, family functioning, and consistent parenting (in that order) in a serial mediation model.	SSS
**LOBODA ET AL. (2016)**	314, 6.2 years ± 4.7 months	Composite SES	Permissive/inconsistent parenting style, highly demanding parenting style, highly responsive parenting style, frequency of stimulating activities	Language proficiency	Permissive/inconsistent parenting style mediated the association between low composite SES and lower child language proficiency. In non-migrant families the mediation occurred through both permissive inconsistent parenting and frequency of stimulating activities.	SSS
**LURIE ET AL. (2021)**	101, 5.55 years ± 0.38	Income to needs ratio, caregiver education	Cognitive stimulation	Receptive language, expressive language quality, expressive language complexity	Income-to-needs ratio and caregiver education were positively associated with cognitive stimulation which in turn mediated the positive association of income to needs ratio and caregiver education with receptive language and expressive language complexity.	Stimulation
**MCCOY, ZUILKOWSKI ET AL. (2015)**	2,711, 6.21-years-old	Wealth, caregiver education	Home-based cognitive stimulation, years of early childhood education, physical development (i.e., height for age)	Receptive language	Wealth was positively associated with home based cognitive stimulation, which in turn mediated the positive association of wealth with language ability. In the multiple mediation models including physical development, wealth was positively associated with physical development via home-based cognitive stimulation and years of early childhood education (in separate paths). These paths mediated the association of wealth with language ability.	Stimulation, Other

AUTHOR, DATE	SAMPLE INFORMATION (N, AGE RANGE/MEAN AGE + SD FOR LANGUAGE ABILITY MEASUREMENT)	SES MEASURE	MEDIATOR	OUTCOME	MAIN FINDINGS	KEY DIMENSION
**MCCOY, CONNERS ET AL. (2015)**	1,904, 3.5 years ± 0.59	Neighborhood disadvantage	Classroom structural quality, positive teacher-child interactions, negative teacher-child interactions	Early literacy skills	NS	
**MCNALLY ET AL. (2019)**	8,062, ~3 years old	Maternal education	Planned pregnancy, smoking during pregnancy, alcohol during pregnancy, born after 36 weeks of gestation, number of previous live births, birth weight, breastfed, mother's age, single parenthood, rarely/never talks to infant, number of days read to child, more than 30 books in the home, regular childcare as infant	Vocabulary	Maternal education was associated with family size, number of days read to the child, and books in the home, which mediated the positive association between maternal education and vocabulary.	Stimulation, Other
**MENDIVE ET AL. (2016)**	989, 52.3 months ± 3.82	Maternal education	Exposure to texts at home, teaching the child to read and write, non-present talk	Picture vocabulary, letter-word identification, dictation	Maternal education was positively associated with exposure to texts at home, which in turn mediated the positive association of maternal education with picture vocabulary and letter-word identification. Teaching the child to read and write mediated the positive association between maternal education and picture vocabulary, letter word identification, and dictation.	Stimulation
**NATALE ET AL. (2021)**	1,157, ~4 years old	Composite SES	Duration of breastfeeding	Child vocabulary	Low SES was associated with shorter duration of breastfeeding, which in turn mediated the association between low SES and lower child vocabulary.	Other
**RAVIV ET AL. (2004)**	1,016, ~36 months	Income to needs ratio, maternal education	Maternal sensitivity, home cognitive stimulation	Verbal comprehension, expressive language, receptive verbal conceptual skills	A higher income-to-needs ratio and higher maternal education were positively associated with maternal sensitivity and home cognitive stimulation. In turn, these associations mediated the positive association between SES and verbal comprehension, expressive language, and receptive verbal conceptual skills.	Support, Stimulation

AUTHOR, DATE	SAMPLE INFORMATION (N, AGE RANGE/MEAN AGE + SD FOR LANGUAGE ABILITY MEASUREMENT)	SES MEASURE	MEDIATOR	OUTCOME	MAIN FINDINGS	KEY DIMENSION
**RUBIO-CODINA ET AL. (2016)**	1,533, 6-42 months	Neighborhood SES	Firstborn child, height-for-age, home environment	Receptive language, expressive language	Neighborhood SES was positively associated with height-for-age and quality of the home environment. These variables mediated the positive association of neighborhood SES with receptive and expressive language.	SSS, Other
**SINGH ET AL. (2022)**	902, 21.3 months ± 5.15	Household income, maternal education, paternal education	Child reading activities, non-dominant language exposure	Dominant language size, conceptual vocabulary size, non-dominant vocabulary size	Higher maternal education and household income were associated with higher levels of reading activities, which led to higher dominant language vocabulary sizes. Higher maternal and paternal education were associated with higher levels of reading activities, which led to higher conceptual vocabulary sizes. However, as paternal education increased, reading behaviors increased, but this was further associated with reduced non-dominant language exposure, which was associated with lower non-dominant language vocabulary sizes.	Stimulation
**SPEYBROECK ET AL. (2012)**	3,949, ~5.9 years	Composite SES	Teacher's expectations	Language achievement	Higher SES was associated with higher teachers' expectations, which in turn mediated the positive association between SES and language achievement. The effect for math achievement was slightly stronger for majority ethnic than for minority ethnic children.	Other
**SWANSON ET AL. (2019)**	96, ~24 months	Maternal education	Language exposure (adult word count), caregiver-child interactions (conversational turn count)	Child language ability	Maternal education was positively associated with adult word count and conversational turn count, which mediated the positive association between maternal education and child language ability.	Stimulation
**WEI ET AL. (2021)**	955, 251 teachers in 159 preschools, 4.19 years ± 0.46	Neighborhood SES, neighborhood resources	Preschool classroom quality: teacher emotional support, instructional support, classroom organization	Language ability and literacy	While the total indirect effect was significant when all three mediators were included in the model simultaneously, no indirect effects of specific mediators were significant.	Other

Table S4. Moderation findings for language ability

AUTHOR, DATE	SAMPLE INFORMATION (N, AGE RANGE/MEAN AGE + SD)	SES MEASURE	MODERATOR	OUTCOME	MAIN FINDINGS	KEY DIMENSION
**BAYDAR & AKCINAR (2015)**	902, 36 - 47 months	Maternal education, composite economic status (based on household goods, quality of the physical environment, material possessions, household expenditures)	Maternal responsiveness, support from the neighbors	Receptive vocabulary	Responsiveness and vocabulary were more strongly positively associated for the children from low SES backgrounds than for other children.	Support
**DEARING ET AL. (2009)**	1,364, 54 months, 1st, 3rd, and 5th grade	Income-to-needs ratio	Childcare quality	Picture vocabulary (average and across all four time points)	NS	SSS
**GEOFFROY ET AL. (2007)**	2,297, 55.4 months ± 4.5	Composite SES	Nonmaternal care	Receptive vocabulary	Full-time nonmaternal care in the first year of life was associated with higher receptive vocabulary scores among children from low SES families, but not among children from adequate SES families.	SSS
**LUO ET AL. (2022)**	165, ~36 months	Income-to-needs ratio, parent education	Maternal referential question asking	Receptive language, productive vocabulary, expressive language	Referential questions (but not Y/N or advanced questions) were associated with receptive language outcomes for children from low and middle education, but not high education backgrounds.	Stimulation

Table S5. Mediation findings for academic achievement

AUTHOR, DATE	SAMPLE INFORMATION (N, AGE RANGE/MEAN AGE + SD FOR AA MEASUREMENT)	SES MEASURE	MEDIATOR	OUTCOME	MAIN FINDINGS	KEY DIMENSION
**AGIRDAG (2018)**	T1 = 1,761, T2 = 1,643, T1 (8-9 years) and T2 (9-10)	School SES	Teachability culture (beliefs regarding the teachability of their pupils)	Science achievement and achievement growth	Higher school SES was associated with higher teachability culture, which in turn mediated the positive association between school SES and science achievement. NS for growth.	Other
**AINSWORTH (2002)**	13,196, 10th grade	Neighborhood SES	Collective socialization (time spent doing homework and educational expectations), effective social control (how many friends have dropped out without graduating), social capital operationalized as intergenerational closure (how many of youth's friends parent knows), perceptions of occupational opportunities (occupational expectations), school characteristics (school atmosphere)	Math/reading composite score	Higher neighborhood SES was associated with higher collective socialization, effective social control, social capital, occupational expectations, and higher quality school atmosphere. These variables mediated the positive association between neighborhood SES and math/reading composite scores.	Other, BCF
**ALBERT ET AL. (2020)**	203, 13.2 years ± 0.4	Composite SES	Verbal working memory, spatial working memory, response inhibition, strategic planning, verbal fluency	Math and reading achievement	Higher SES was associated with improved verbal working memory and strategic planning, which mediated the positive association between SES and math achievement. Higher SES was also associated with improved working memory and verbal fluency, which mediated the positive association between SES and reading achievement.	CC
**ALTSCHUL (2012)**	1,609, 14.3 years ± 0.62	Family income, mothers education, fathers education, mothers occupation, fathers occupation	Discussion of school related issues between parents and students, parental homework help, parental involvement with school organizations, educational resources in the home, allocation of resources to out-of-school instruction, parental involvement in enriching activities	Composite academic achievement	Family income and maternal education were positively associated with educational resources in the home and allocation of resources to out of school instruction, which mediated the positive association of family income and maternal education with academic achievement.	Stimulation
**BACHMAN ET AL. (2022)**	149, 4.44 years ± 0.30	Composite SES	Composite EF	Math achievement, number skills, ANS acuity (spatial skills and children's intuitive sense of quantities)	Higher SES was associated with EF, which in turn mediated the association between SES and math achievement, number skills, and ANS acuity.	CC

AUTHOR, DATE	SAMPLE INFORMATION (N, AGE RANGE/MEAN AGE + SD FOR AA MEASUREMENT)	SES MEASURE	MEDIATOR	OUTCOME	MAIN FINDINGS	KEY DIMENSION
**BAKER ET AL. (2018)**	7,700, 50.84 months ± 4.04	Poverty status	Paternal warmth, maternal warmth, paternal home learning stimulation, maternal home learning stimulation	Reading achievement, math achievement	Not living in poverty was associated with higher levels of paternal warmth and maternal home learning stimulation, which mediated the association between poverty status and reading and math achievement. Not living in poverty was also associated with higher levels of paternal home learning stimulation which mediated the association between poverty status and reading achievement.	Support, Stimulation
**BARNES ET AL (2022)**	3,072, 1st grade	Composite SES	Working memory, cognitive flexibility	Reading achievement	Higher SES was associated with higher working memory and increased cognitive flexibility, which in turn mediated the positive association between SES and reading achievement.	CC
**BARR (2015)**	8,650, T1 (9th grade) and T2 (11th grade)	Composite SES	Student and parent physical health problems	Change in math achievement	Higher SES was associated with lower instances of student and parent health problems which mediated the positive association between SES and growth in math achievement.	Other
**BETANCUR ET AL. (2018)**	9,250, T1: 3rd grade (111.0 months ± 4.4), T2: 5th grade (134.6 months ± 4.5), T3: 8th grade (171.3 months ± 4.4)	Income, parental education	In-home science learning opportunities, out of home science learning opportunities, school-level variables (science instructional time, science specific teacher training, years of teaching science), prior science skills, prior reading skills, prior math skills	Science achievement	Higher income and parental education were associated with higher out-of-home science opportunities, higher number of years of teaching science, prior science skills, prior reading skills and prior math skills. These variables mediated the positive association of income and parental education with science achievement. Higher education was associated with higher in-home science opportunities, which mediated the positive association between education and science achievement. Results were consistent for the outcome across grades.	Stimulation, CC, Other
**BLAKEY ET AL. (2020)**	175, 48 months (39–54 months)	Neighborhood SES	Composite EF	Mathematical ability	Higher SES was associated with higher EF which in turn mediated the positive association between SES and math ability.	CC
**BODOVSKI & FARKAS (2008)**	8,035, 86 months ± 4.35	Composite SES	Parental educational expectations, concerted cultivation, approaches to learning	Reading Achievement	High SES was associated with higher levels of parent educational expectations, higher concerted cultivation, and more positive learning behaviors. These variables (in that order) mediated the positive association between SES and reading achievement.	Other, Stimulation
**CADIMA ET AL. (2015)**	186, 5.1 years ± 6.7	Composite SES	Behavioral regulation	Letter identification/literacy, math ability	SES was positively associated with behavioral regulation which in turn mediated the positive association between SES and mathematical ability.	CC

AUTHOR, DATE	SAMPLE INFORMATION (N, AGE RANGE/MEAN AGE + SD FOR AA MEASUREMENT)	SES MEASURE	MEDIATOR	OUTCOME	MAIN FINDINGS	KEY DIMENSION
**CALLAN ET AL. (2017)**	475,460, 15 years	Composite SES, school SES	Learning approaches: metacognitive summarizing, metacognitive understanding and remembering, control, elaboration, memorization	Math achievement, reading achievement, science achievement	Higher family and school SES were associated with higher use of metacognitive summarizing, metacognitive understanding, remembering, and control. These variables mediated the positive association of family and school SES with math, reading, and science achievement.	CC
**CAROLAN (2015)**	10,305, 11th grade	Composite SES	Concerted cultivation, close friend behaviors, parental expectations, math identity	Math achievement	Higher SES was positively associated with increased parental expectations, which in turn mediated the positive association between SES and math achievement. In addition, higher concerted cultivation, and higher math identity (in that order) mediated the association between composite SES and math achievement.	Stimulation, Other
**CASCELLA (2020)**	422,865, 15 years	Composite SES	Reading ability	Math ability	Higher SES was associated with higher reading ability which in turn mediated the positive association between SES and math ability.	CC
**CHEN AT AL. (2018)**	2,294, 8th grade	Composite SES	Parent-child relationship	Reading achievement	SES was positively associated parent-child relationship quality, which in turn mediated the positive association between SES and reading achievement.	Support
**CHENG & WU (2017)**	149, 6.25 years ± 0.34	Composite SES	Vocabulary knowledge, morphological awareness	Reading comprehension	Higher SES was associated with higher levels of vocabulary knowledge and morphological awareness, which (in that order) mediated the positive association between SES and reading comprehension.	CC
**CHEVALÈRE ET AL. (2022)**	2,379, 14.6 years ± 1.52	Parent occupation	Working memory processing, working memory storage, academic self-concept	Academic achievement	Higher SES was associated with higher working memory processing and academic self-concept. These variables mediated the positive association between SES and academic achievement.	CC, Other
**CHIEN & MISTRY (2013)**	17,565, 86.92 months ± 4.27	Income-to-needs ratio	Material hardship, parent stress, parenting practices (parental warmth, physical punishment, cognitive stimulation, and rules and routines), parental investment (involvement in school, extracurricular activities, books in the home), school resources	Academic achievement	Higher SES was associated with lower material hardship and parent stress, parenting practices characterized by higher parental warmth, less physical punishment, and more cognitive stimulation, more parental investment and more school resources. These variables mediated the positive association between SES and academic achievement.	Stress, SSS, Stimulation, BCF
**COLEY ET AL. (2019)**	3,396, data from two longitudinal studies between preschool and kindergarten years	Classroom economic composition	Peer cognitive skills, instructional quality	Math achievement, reading skills	Classroom economic composition was positively associated with peer reading skills, which mediated the association between classroom SES and reading skills.	Other
**CROOK & EVANS (2014)**	1,009, 5th grade	Income-to-needs ratio	Planning	Math achievement, reading achievement	Income-to-needs ratio was positively associated with planning abilities, which mediated the positive association between income-to-needs and math achievement.	CC

AUTHOR, DATE	SAMPLE INFORMATION (N, AGE RANGE/MEAN AGE + SD FOR AA MEASUREMENT)	SES MEASURE	MEDIATOR	OUTCOME	MAIN FINDINGS	KEY DIMENSION
**CROSNOE & COOPER (2010)**	17,401, T1 (kindergarten, 5.70 years ± 0.36) and T2 (1st grade)	Composite SES	Parenting stress, parent depression, child externalizing problems, child internalizing problems, organized activities, school-based parental involvement, cognitively stimulating materials, rules and routines	Change in math ability, change in reading ability	Higher SES was associated with lower parent stress, lower child externalizing and internalizing problems, higher organized activities, higher school-based parental involvement, and greater availability of cognitively stimulating materials. These variables mediated the positive association between SES and change in math ability. Higher SES was associated with lower parent depression, lower child externalizing and internalizing problems, increased organized activities, more cognitively stimulating materials, and more rules and routines. These variables in turn mediated the positive association between SES and change in reading ability.	Stress, SSS, CC, Stimulation, Other
**DILWORTH-BART (2012)**	49, 54-66 months	Composite SES	Composite EF	Math achievement, literacy achievement, knowledge of story-and-print concepts	Higher SES was associated with higher EF, which in turn mediated the positive association between SES and math achievement.	CC
**DOLEAN ET AL. (2019)**	500, T1= 7.07 years ± 0.48, T2 (6 months after T1), T3 (6 months after T2) and T4 (6 months after T3)	Composite SES	Rapid automatized naming, phonological awareness, letter knowledge, nonverbal IQ, school absenteeism	Nonword reading initial status, nonword reading growth	Higher SES was associated with more rapid automatized naming, higher phonological awareness, letter knowledge, and nonverbal IQ, and lower school absenteeism. These variables mediated the positive association between SES and nonword reading initial status and nonword reading growth.	CC, Other
**DUPÉRÉ ET AL. (2010)**	1,364, 1st grade	Neighborhood advantage	Home environment quality, childcare environment quality, classroom environment, school advantage, maternal depression	Reading achievement	Higher neighborhood advantage was associated with higher quality home and childcare environment, as well as higher school advantage, which in turn mediated the positive association between neighborhood advantage and reading achievement.	SSS, Other, BCF
**EAMON (2002)**	1,324, 12.91 years ± 0.74	Poverty	Cognitive home environment, emotional home environment, school behavior problems	Math achievement, reading achievement	Poverty was associated with less cognitively stimulating and emotionally supportive home environments, which were in turn associated with more school behavior problems. These variables in turn mediated the association between poverty and lower math and reading achievement.	Stimulation, Support, CC
**ELLEFSON ET AL. (2020)**	835, 371 from Hong Kong (12.21 years ± 0.99 years) and 464 from UK (11.92 years ± 0.93 years)	Composite SES	Composite EF	Numeracy skills	Higher SES was associated with higher EF performance which in turn mediated the positive association between SES and numeracy skills (only for the U.K male sample).	CC

AUTHOR, DATE	SAMPLE INFORMATION (N, AGE RANGE/MEAN AGE + SD FOR AA MEASUREMENT)	SES MEASURE	MEDIATOR	OUTCOME	MAIN FINDINGS	KEY DIMENSION
**FITZPATRICK ET AL. (2014)**	266, 56.88 months ± 9.06	Preschool SES	General intelligence, composite EF, vocabulary	Applied problems, letter-word identification, picture vocabulary	Higher preschool SES was associated with higher EF, which mediated the positive association between preschool SES and applied problems, letter-word identification, and picture vocabulary performance. Higher vocabulary mediated the positive association between preschool SES and higher scores on applied problems and letter-word identification.	CC
**FORGET-DUBOIS ET AL. (2009)**	693, ~63 months	Composite SES	Exposure to reading, expressive vocabulary	School readiness	Higher SES was associated with exposure to higher reading and expressive vocabulary, which in turn mediated the association between SES and school readiness.	Stimulation, CC
**FUNG & CHUNG (2019)**	109, male mean age 59.98 months, female mean age 58.58 months	Composite SES	Vocabulary knowledge, phonological knowledge	Chinese word reading	NS	
**GALINDO & SONNENSCHEIN (2015)**	19,280, kindergarten age	Composite SES	Math proficiency at kindergarten entry, learning tools (i.e., number of books and CDs, records, and tapes in the home, and whether the child had a computer), parental involvement in school, general learning activities, reading activities, further educational expectations, current educational expectations	Math achievement	Higher SES was associated with higher levels of math proficiency at kindergarten entry, learning tools, parental involvement in school, general learning activities, reading activities, and future educational expectations. These variables in turn mediated the positive association between SES and math achievement for all quintiles of SES.	Stimulation
**GARRETT-PETERS ET AL. (2016)**	1,292, kindergarten year	Income-to-needs	Household disorganization, household instability	Academic achievement	A higher the income-to-needs ratio was associated with lower household disorganization, which in turn mediated the association between income-to-needs and academic achievement.	Stress
**GREENFADER (2019)**	4,360, 66.87 months ± 4.29	Composite SES	English oral language, working memory, cognitive flexibility, inhibitory control	Reading achievement, math achievement	Higher SES was associated with higher English oral language ability and higher working memory, which mediated the association of SES with reading and math achievement. In addition, higher inhibitory control mediated the positive association of SES and reading achievement.	CC
**HAMILTON ET AL. (2016)**	188, 78.99 months ± 4.33	Composite SES	Storybook exposure, literacy teaching, precursor skills (phoneme awareness, emergent decoding, oral language)	Word-level literacy, reading comprehension	The association of SES with word-level literacy and reading comprehension was mediated by storybook exposure and precursor skills such as oral language, emergent decoding, and phoneme awareness.	Stimulation

AUTHOR, DATE	SAMPLE INFORMATION (N, AGE RANGE/MEAN AGE + SD FOR AA MEASUREMENT)	SES MEASURE	MEDIATOR	OUTCOME	MAIN FINDINGS	KEY DIMENSION
**IRUKA ET AL. (2014)**	9,550, 52.95 months ± 4.18	Family income, maternal education	Availability of learning materials, language stimulation, outdoor activities	Literacy achievement, numeracy achievement	Higher family income and higher maternal education were associated with increased language stimulation and outdoor activities. These variables in turn mediated the positive association of higher family income and higher maternal education with literacy and math achievement. Higher family income was also associated with greater availability of learning materials and this in turn mediated the positive association between income and literacy achievement (findings differed between races: Asians, African Americans, Euro-Americans and Hispanics). In general, language stimulation and outside activities were the most consistent mediators for Euro-Americans, learning materials was the most consistent mediator for African Americans, learning materials and language stimulation were the most consistent mediators for Hispanics, and learning materials and outside activities were the most consistent mediators for Asians.	Stimulation
**KORZENIOWSKI ET AL. (2016)**	178, 7.24 years ± 1.17	Composite SES	Composite EF	Academic achievement	SES was positively associated with EF which in turn mediated the positive association between SES and academic achievement.	CC
**KRIEGBAUM & SPINATH (2016)**	6,020, 15.5 years ± 0.55	Father occupation, mother occupation, composite SES	Motivation (math-specific self-concept, task-specific self-efficacy, global self-efficacy, interest; considered separately), composite cognition (intelligence), prior achievement.	Mathematical competence	Intelligence and prior achievement mediated the association of father's SES, mother's SES, and composite SES with mathematical competence. Mother's SES was associated with math-specific self-concept and task-specific self-efficacy, which mediated the positive association between mother's SES and mathematical competence. Math-specific self-concept, task-specific self-efficacy, and interest mediated the association of father's SES with mathematical competence. All motivation variables mediated the association of composite SES with mathematical competence.	CC

AUTHOR, DATE	SAMPLE INFORMATION (N, AGE RANGE/MEAN AGE + SD FOR AA MEASUREMENT)	SES MEASURE	MEDIATOR	OUTCOME	MAIN FINDINGS	KEY DIMENSION
**LARSON ET AL. (2015)**	6,600, kindergarten entry	Composite SES	Mothers age, child's age, family structure, household size, mothers reported pre-pregnancy BMI, birthweight, child's global health, parent reading, home computer, number of children's books, positive parenting interactions, rules about food, high expectations for child's educational attainment, preschool attendance, participation in organized classes/activities, parent supportiveness	Reading ability, math ability	Higher SES was associated with older child age, lower pre-pregnancy BMI of mother and household size, higher birthweight, better child health, higher levels of parent reading, home computer, higher levels of positive interactions with parents, more rules about food, higher parental expectations, and preschool attendance. These variables mediated the positive association between SES and reading ability.<br><br>Higher SES was associated with older child age, lower pre-pregnancy BMI of mother, and maternal depression, higher birthweight, better child health, higher levels of parent reading, home computer, higher levels of positive interactions with parents, parent supportiveness, more rules about food, higher parental expectations, and preschool attendance. These variables mediated the positive association between SES and math ability.	Other, CC, Stimulation, Support, SSS
**LAWSON & FARAH (2015)**	336, T1: 10.13 years ± 2.94, T2: 2-years later	Composite SES	Composite EF, verbal memory	Change in calculation, change in passage comprehension	High SES was associated with higher EF performance, which in turn mediated the positive association between SES and change in math achievement.	CC

AUTHOR, DATE	SAMPLE INFORMATION (N, AGE RANGE/MEAN AGE + SD FOR AA MEASUREMENT)	SES MEASURE	MEDIATOR	OUTCOME	MAIN FINDINGS	KEY DIMENSION
**LEI (2018)**	1,278 children, 10-15 years	Composite neighborhood SES	Neighborhood institutional resources (facilities), neighborhood institutional resources (extra-curricular activities), neighborhood institutional resources (key school i.e., schools that have higher-quality teachers and infrastructure), collective socialization (educational aspiration), peer contagion (best friend's deviant behaviors, neighborhood deviant behaviors), neighborhood social organization (social capital, household property victimization, physical assault victimization), neighborhood physical environment (pollution, built environment)	Verbal scores, math achievement	Higher neighborhood SES was positively greater neighborhood institutional resources (availability of extra-curricular activities, key schools), collective socialization (higher levels of educational aspirations), and higher social organization (i.e., lower household property victimization), which mediated the association of SES with verbal achievement. In a multiple mediation model, the effects of extra-curricular activities and household property victimization were no longer significant. In addition, children with no best friend perform significantly worse than the other children. For math achievement, higher neighborhood institutional resources (availability of extra-curricular activities), collective socialization (higher levels of educational aspirations), neighborhood social capital, and higher social organization (i.e., lower physical assault) mediated the association of SES with math achievement. In a multiple mediation model, the effects of extra-curricular activities were no longer significant. In addition, having one or more friends with deviant behaviors was associated with worse performance.	BCF, Other
**LIU ET AL. (2015)**	199, T1: 58.02 months ± 3.74, T2: 1-year later	Composite SES	Phonological awareness, phonological memory, working memory, Chinese vocabulary, English vocabulary	K2 Chinese word reading, K2 English word reading, K3 Chinese word reading, K3 English word reading	Higher SES was associated with higher phonological awareness in K2, which in turn mediated the positive relationship between SES and both K2 Chinese word reading and K2 English word reading. Higher SES was associated with higher English vocabulary in K2 and K3, which in turn mediated the positive association of SES with K2 and K3 English word reading.<br><br>The association between SES and K3 Chinese word reading and English word reading was mediated by K2 phonological awareness. The association between SES and K3 English word reading was mediated by K2 vocabulary.	CC
**LONG & PANG (2016)**	5,066, 15–17 years old	Parental education, family wealth	Parental expectations	Mathematics achievement, problem-solving achievement	Higher parental education was associated with higher parental expectations which mediated the positive association between SES and both mathematics and problem-solving achievement. Higher family wealth was associated with lower parental expectations, thus acting as a suppressor in the association of SES and mathematics and problem-solving achievement.	Other

AUTHOR, DATE	SAMPLE INFORMATION (N, AGE RANGE/MEAN AGE + SD FOR AA MEASUREMENT)	SES MEASURE	MEDIATOR	OUTCOME	MAIN FINDINGS	KEY DIMENSION
**LURIE ET AL. (2021)**	77, 7 years ± 0.46	Income-to-needs, caregiver education	Receptive language, expressive language quality, expressive language complexity	Academic achievement	Higher income-to-needs ratio was associated with higher receptive language ability which in turn mediated the association between income-to-needs ratio and academic achievement. Higher caregiver education was associated with higher receptive language ability and higher expressive language quality which in turn mediated the association between caregiver education and academic achievement.	CC
**MARKS ET AL. (2006)**	172,000, 15 years, PISA sample of students in over 6,000 schools across 32 countries	Composite SES	Material resources, social resources, cultural resources, educational differentiation—school tracks and school types, and curriculum tracking within schools, operationalized as academic location (the position or stage within the educational system that a student occupies, determined by both their grade level and the specific school program they are enrolled in), individual schools (specific schools unique characteristics such as specialized programs, demographics, and educational approaches)	Reading achievement, math achievement, science achievement	Higher SES was associated with both greater material resources and greater cultural resources, which in turn mediated the positive association between SES and reading, math, and science achievement (results varied based on country). In many countries, educational differentiation mediated the relationship between SES and achievement. Countries with highly tracked systems tend to show stronger relationships. In a few countries, higher SES was associated with greater social resources and this in turn mediated the positive association between SES and reading, math, and science achievement.	Support, Stimulation
**MCCOY, CONNERS ET AL. (2015)**	1,904, 3.5 years ± 0.59	Neighborhood disadvantage	Classroom structural quality, positive teacher-child interactions, negative teacher-child interactions	Early math skills, early literacy skills	NS	
**MERZ ET AL. (2014)**	308, 3.21 ± 0.54	Parent education	Effortful control	Early math skills, early literacy	Higher effortful control mediated the positive association between parent educational attainment and early math and literacy skills	CC
**MISTRY ET AL. (2004)**	1,363, ~36 months	Income-to-needs ratio	Perceived financial adequacy, maternal depression, maternal sensitivity	School readiness	For families living at the poverty threshold, family processes (perceived financial adequacy -> maternal depression -> maternal sensitivity) mediated the association of average income with school readiness skills. The strength of these associations diminished as average income exceeded the poverty threshold.	SSS

AUTHOR, DATE	SAMPLE INFORMATION (N, AGE RANGE/MEAN AGE + SD FOR AA MEASUREMENT)	SES MEASURE	MEDIATOR	OUTCOME	MAIN FINDINGS	KEY DIMENSION
**MURPHY ET AL. (2022)**	1,227, 1st grade	Income-to-needs ratio	EF before school entry	Academic achievement	Higher family income was related to higher levels of academic skills through higher EF before school entry. A three-way interaction between income, parenting, and learning materials indicated that positive parenting and learning materials buffered the association between low income and lower EF, and subsequently academic outcomes in 1st grade.	CC
**MYRBERG & ROSÉN (2008)**	Between ~3000 and 6000 in each country, 4th grade (9-10 years), samples from 7 countries	Parental education	Home library, early reading activities, early reading activities	Reading achievement	In all countries, higher parental education was associated with larger home libraries, more early reading activities, and higher early reading ability. This in turn mediated the positive association between parental education and reading achievement. However, the extent to which the size of the home library, early reading activities, and early reading abilities mediated this association varied from country to country.	Stimulation
**MYRBERG & ROSEN (2009)**	10,632, 3rd grade (8 years)	Parent education	Number of books at home, early reading activities, early reading abilities	Reading achievement	Higher parent education was associated with more books at home, more reading activities and higher early reading abilities, which in turn mediated the positive association between parent education and reading achievement.	Stimulation
**NESBITT ET AL. (2013)**	206, 78.62 months ± 5.66	Composite SES	EF, expressive vocabulary	Math achievement, literacy achievement	High SES was associated with higher EF performance which in turn mediated the positive association between SES and math and literacy achievement.	CC
**PERRY ET AL. (2017)**	546, ~16.5 years	Neighborhood SES	Academic self-efficacy, social self-efficacy, emotional self-efficacy	Academic achievement	Higher SES was associated with higher academic self-efficacy which mediated the positive association between SES and academic achievement.	Other
**PERRY ET AL. (2018)**	1,044, 2nd grade	Composite SES (family income-to needs ratio, household density, neighborhood safety, maternal education, consistent partnership of a spouse/partner living in the home, maximum work hours of primary or secondary caregiver per week, and job prestige)	EF	Academic achievement: reading and math	Low SES was associated with lower executive function which mediated the association between low SES and lower academic performance.	CC

AUTHOR, DATE	SAMPLE INFORMATION (N, AGE RANGE/MEAN AGE + SD FOR AA MEASUREMENT)	SES MEASURE	MEDIATOR	OUTCOME	MAIN FINDINGS	KEY DIMENSION
**REN ET AL. (2020)**	321, 11.73 years	Composite SES	Adolescent own educational expectations	Academic achievement	Higher SES was associated with higher educational expectations which in turn mediated the positive association between SES and academic achievement. In addition, subjective SES significantly moderated the pathway from SES to educational expectations (but not to academic achievement) such that the relationship between SES and educational expectations was significant among adolescents with lower but not higher levels of subjective SES. The moderated mediation model indicated that adolescents with low subjective SES, the indirect effect of SES on academic achievement through educational expectations was significant. However, this indirect effect was not significant for adolescents with high subjective SES.	Other
**RJOSK ET AL. (2014)**	8,047, ~15 years	Classroom SES composition	Instructional quality	Reading achievement	NS	
**ROSEN ET AL. (2019)**	101, outcomes measured at T2 (~7 years)	Income-to-needs ratio, maternal education	Working memory, cognitive flexibility, inhibition	Academic achievement	A higher income-to-needs ratio and maternal education was associated with higher working memory and cognitive flexibility. This in turn mediated the positive association of income-to-needs and maternal education with academic achievement.	CC
**SCHNEIDER & MAGUIRE (2022)**	240, 137.73 months ± 26.46	Maternal education	Receptive vocabulary knowledge, reading comprehension, working memory, phonological memory	Inferring word meaning task	Higher maternal education was associated with higher receptive vocabulary knowledge and higher reading comprehension, which in turn mediated the positive association between maternal education and number of words correctly inferred.	CC
**SLUSSER ET AL. (2019)**	86, 5.1 years	Parent education	Number word knowledge, general vocabulary	Math ability	Higher parental education was associated with higher number word knowledge and higher general vocabulary, which in turn mediated the positive association between parent education and math ability.	CC
**SPEYBROECK ET AL. (2012)**	3,949, 5.9 years	Composite SES	Teacher's expectations	Math achievement	Higher SES was associated with higher teachers' expectations, which in turn mediated the positive association between SES and language achievement. The effect for math achievement was slightly stronger for majority ethnic than for minority ethnic children.	Other
**SUN ET AL. (2018)**	3,331 (across 3 countries - Cambodia, Mongolia, and Vanuatu), 36-71 months	Composite SES	Preschool attendance, composite EF	Language achievement, math achievement, literacy achievement	Higher SES was associated with higher preschool attendance and composite EF, which in turn mediated the positive association between SES and language, math, and literacy achievement. However, results varied by country.	Other, CC

AUTHOR, DATE	SAMPLE INFORMATION (N, AGE RANGE/MEAN AGE + SD FOR AA MEASUREMENT)	SES MEASURE	MEDIATOR	OUTCOME	MAIN FINDINGS	KEY DIMENSION
**TOMASZEWSKI ET AL. (2020)**	3,215, 12-13 years	Composite SES	Student engagement	Reading achievement, numeracy achievement	Higher SES was associated with higher student engagement which in turn mediated the positive association between both SES and reading and numeracy achievement.	Other
**WATERS ET AL. (2021)**	1,273, T1: 4.64 years ± 0.09, T2: 1st grade	Parent education, income-to-needs ratio	Attention control, response inhibition, working memory	Math achievement, reading achievement, and change over time	Higher parental education was associated with higher attention control and working memory, which in turn mediated the positive association between parent education and reading achievement. A higher income-to-needs ratio was associated with higher working memory and this in turn mediated the positive association between income-to-needs and math achievement. Working memory also mediated the association of parent education with change in math achievement.	CC
**WOLF & MCCOY (2017)**	2,137, 5.2 years ± 1.34	Household wealth, caregiver education level	At home stimulation, caregiver school involvement, number of books in the household	Numeracy achievement, literacy achievement	Higher household wealth and caregiver education were associated with higher levels of at-home stimulation and caregiver school involvement, which mediated the positive association between household wealth/caregiver education and both numeracy and literacy achievement.	Stimulation, Other
**XUAN ET AL. (2019)**	10,784, 14.52 years ± 1.11 (7th grade-9th grade)	School SES	Teacher-student relationship quality	Math achievement	School SES was positively associated with teacher-student relationship quality, which in turn mediated the positive relationship between school SES and math achievement.	Support
**YE ET AL. (2021)**	2,686, T1 (2014): 12.42 years ± 1.75, T2: 2016, T3: 2018	Composite SES	Frequency of internet use to study, socialize, entertain, and do commercial activities	Math achievement	High SES was associated with higher frequency of internet use for studying which in turn mediated the positive association between SES and math achievement. These findings were only significant later in adolescence.	Other
**YEUNG ET AL. (2022)**	26,281, 15.78 years ± 0.29	Composite SES and average SES at the school-level	Student level expectancy, student level value, school level expectancy and school level value	Reading achievement	Higher SES was associated with higher student level expectancy and student level value, which in turn mediated the positive association between SES and reading achievement. Higher school-level SES was associated with higher school level expectancy and higher school level value (only in mainland China and not other regions). These variables mediated the positive association between SES and reading achievement but in opposite directions; there was a suppressor effect of school-level expectancy.	Other
**YU & HANNUM (2007)**	2,000, 11 years ± 1.1	Wealth, household expenditures	Nutritional environment	Language (Chinese) test scores, math test scores	High SES (for both indicators) was associated with higher quality of the nutritional environment. This in turn mediated the positive association of SES with language (Chinese) and mathematics scores.	Other

AUTHOR, DATE	SAMPLE INFORMATION (N, AGE RANGE/MEAN AGE + SD FOR AA MEASUREMENT)	SES MEASURE	MEDIATOR	OUTCOME	MAIN FINDINGS	KEY DIMENSION
**ZHANG ET AL. (2013)**	262, 3rd grade (~9 years)	Composite SES	Phonology, vocabulary, phonology -> vocabulary, vocabulary -> phonology	Chinese reading	Higher SES was associated with higher phonological abilities and vocabulary which in turn mediated the positive association between SES and Chinese character recognition.	CC
**ZHANG ET AL. (2019)**	588, ~ 5.5 years and 6 months later	Composite SES	Receptive vocabulary, behavioral regulation	Chinese reading, math achievement, life science, and earth and physical science	The association of high SES with higher Chinese reading ability, and achievement in math, life science, and earth and physical sciences was mediated by behavioral regulation and receptive vocabulary across timepoints in a cross-lagged panel model framework.	CC

Table S6. Moderation findings for academic achievement

AUTHOR, DATE	SAMPLE INFORMATION (N, AGE RANGE/MEAN AGE + SD)	SES MEASURE	MODERATOR	OUTCOME	MAIN FINDINGS	KEY DIMENSION
**BEISLY ET AL (2020)**	179, 50.03 months ± 8.79	Composite SES	Children's learning behaviors (attention/persistence, competence/motivation, and attitude toward learning), executive functioning	Literacy achievement, math achievement	Higher levels of learning behaviors buffered the association between low SES and lower math achievement (i.e., the association between SES and math achievement was more positive at lower levels of learning behaviors)	CC
**BERNARDO ET AL. (2021)**	~6925, ~15	Wealth, household possessions, household SES (based on social, economic, and cultural status)	Growth mindset	Science and mathematics achievement	Growth mindset was positively associated with both science and math achievement for those with high SES but not low SES (for all three indicators).	Other
**BOYES ET AL. (2017)**	3401, 13.45 years ± 2.15	Poverty ((total number of necessities lacking)	Gender	School dropout (defined as being enrolled in school at baseline assessment but no longer enrolled in school at the 1-year follow-up assessment)	At low and mean levels of household poverty, girls were at higher risk of school dropout.	Other
**CASCELLA (2019)**	T1=30,868, T2=38,091, primary school (5th grade) and secondary school (10th grade)	Individual, classroom, and school SES	Gender	Math achievement	Males benefitted more from high SES with regards to primary and secondary school math achievement.	CC
**CHEN AT AL. (2018)**	2,294, 8th grade	Composite SES	Learning motivation	Reading ability	High level of learning motivation buffered the association between low SES and lower reading ability (i.e., the association between SES and reading ability was more positive at lower levels of learning behaviors)	CC
**CLARO ET AL. (2016)**	~168,000, 10th graders	Family income	Fixed vs growth mindset	Math performance, language performance	Students from low-income backgrounds were twice as likely as the highest-income students to report a fixed mindset, and their mindset was a stronger predictor of math and language achievement. Low-income students who held a growth mindset were buffered against the effects of poverty on achievement: students in the lowest 10th percentile of family income who exhibited a growth mindset showed academic performance as high as that of fixed mindset students from the 80th income percentile.	Other

AUTHOR, DATE	SAMPLE INFORMATION (N, AGE RANGE/MEAN AGE + SD)	SES MEASURE	MODERATOR	OUTCOME	MAIN FINDINGS	KEY DIMENSION
**DEARING ET AL. (2009)**	1,364, 54 months, 1st, 3rd, and 5th grade	Income-to-needs ratio	Childcare quality	Reading achievement, math achievement (average of 3rd and 5th grade), applied problems, and letter word identification (average over four time points)	The association between income-to-needs and average achievement for all four outcomes was less steep for children who were in two episodes of higher quality childcare compared with those who were never in higher quality care. There was no significant interaction between higher quality childcare and income-to-needs for linear or quadratic change in achievement, nor did the interactions differ significantly between third and fifth grades for math and reading scores. This suggests that the achievement advantage of attending higher quality childcare for low-income children was stable over time.	Other
**ELLIOTT & BACHMAN (2018)**	13,399, ~6 years	Income, education	Educational expectations, school readiness beliefs	Reading achievement, math achievement	NS	
**FAGAN (2016)**	7,300, 52.8 months ± 4.17	Income	Family structure (single-parent, married-parent, stable-cohabiting households)	Emerging math and literacy skills (composite)	Household income had a stronger positive association with child achievement in married-parent households than in single-parent or stable-cohabiting households.	Other
**GALINDO & SONNENSCHEIN (2015)**	19,280, kindergarten age	Composite SES	Math achievement at kindergarten entry, general learning activities, reading activities	Math achievement	Children from the 5th SES quintiles benefitted more from math proficiency at kindergarten entry as well higher levels of learning and reading activities at home than children from the 4th quintile.	CC, Stimulation
**KING & TRINIDAD (2021)**	15,362, 10th grade	Composite SES, school SES	Growth mindset	Math achievement	Growth mindset was negatively associated with achievement for those with low SES as opposed to a positive association for those with high SES. NS for school SES.	Other
**KOEPP ET AL. (2022)**	5th grade: 2,167,729, 10.89 years ± 1.00<br>9th grade: 1,782,899, 14.96 years ± 1.03	Parent education	Parent involvement in children's education	Math achievement, reading achievement	For fifth graders, parent involvement was positively associated with math and reading achievement for those parents with education ranging from less than a fifth grade through college completion. However, for children whose parents never attended school, parent involvement was not associated with reading and math achievement.	Other
**KRANJAC & KRANJAC (2021)**	13,694, outcomes measured 1st, 3rd, 5th, and 8th grade, 4-15 years	Poverty status based on composite SES	Under-weight, over-weight, obese	Reading achievement, math achievement	Obesity status exacerbates the negative influence of low SES on reading and math achievement. Overweight status exacerbates the negative influence of low SES on reading achievement.	Other
**LAURIN ET AL. (2015)**	1269, ~12 years	Composite SES (divided into low SES and adequate SES)	Trajectories of childcare services (CCS) based on "intensity" (i.e., number of hours per week) and "type" (i.e., center-based vs never center-based CCS) - obtained from 5 months to 4.5 years	Reading achievement, math achievement, writing skills	For low-SES children, exposure to CCS (any type) .35 hours a week was associated with better academic achievement in all disciplines (reading, writing, and mathematics) at 12 years. We also found that low-SES children exposed to center-based CCS earlier in life (i.e., from 5 months) had better reading, writing, and mathematics scores than low-SES children never exposed to center-based CCS, whereas those exposed to center-based CCS later (i.e., from 1.5 years) only had better reading and mathematics scores.	Other

AUTHOR, DATE	SAMPLE INFORMATION (N, AGE RANGE/MEAN AGE + SD)	SES MEASURE	MODERATOR	OUTCOME	MAIN FINDINGS	KEY DIMENSION
**LI ET AL. (2016)**	826, 55.26 months ± 10.28	Preschool SES	Family SES, home learning environment, authoritative parenting	Literacy skills	NS	
**LITTLE ET AL., (2018)**	220 (85 in summer attendance group), kindergarten, first, and second grade	Free/reduced price lunch status	Summer program participation (yes vs no)	Growth in math achievement	NS for moderation (students who did and did not qualify for free or reduced lunch saw similar gains over the summer if they participated in the summer program, indicating that the summer program experience was supportive for students across a range of income backgrounds. Students who participated made moderately larger mathematics achievement gains than students who did not participate).	Stimulation
**LIU ET AL. (2015)**	199, 58.02 months ± 3.74 and 1-year later	Composite SES	Phonological awareness, phonological memory, working memory, Chinese vocabulary, English vocabulary	K2 Chinese word reading, K2 English word reading, K3 Chinese word reading, K3 English word reading	NS	
**LOSIER ET AL. (2022)**	2001, ~20 years	Composite SES	Early care and education exposure (late, early, and never)	High school diploma	NS	
**MCCORMICK ET AL. (2017)**	1,053, 54 months and 1st, 3rd, and 5th grades	Maternal education, family income	Teacher-child conflict, teacher-child closeness	Reading achievement, math achievement	Children from families with low maternal education and higher levels of teacher-child conflict and lower levels of teacher-child closeness exhibited lower reading achievement. The magnitude of the positive association between change in teacher-child closeness and change in reading achievement was larger for children with low maternal education. The strength of the negative association between change in teacher-child conflict and change in reading achievement was also larger for children with low maternal education. The positive association between change in teacher-child closeness and change in math achievement across middle childhood was stronger for children from low-income families, relative to more affluent families.	Stress, Support
**OLSEN & HUANG (2021)**	8,380, 1st grade	Composite SES	Student-teacher closeness	Math achievement	Close student-teacher relationships buffered the association between low SES and lower math achievement (close student-teacher relationships are beneficial for all students but are especially beneficial for students from low SES backgrounds).	Support
**PEARMAN (2020)**	1,076, 3rd grade	Neighborhood poverty	PreK attendance	Reading achievement, math achievement	Among children living in high-poverty neighborhoods, assignment to the PreK attendance group was associated with higher reading achievement.	Other
**RIBNER ET AL. (2017)**	807, 68.9 months ± 4.07	Income-to-needs ratio	Television viewing	Math ability	High levels of TV viewing had a negative association with math ability only at low levels of income-to-needs ratio.	Other
**RICHARDS ET AL. (2015)**	327,698, 6 - 8 years	Neighborhood disadvantage	Preterm birth	Math achievement (pass vs fail)	Neighborhood deprivation was associated with increased risk of mathematics failure for children born preterm.	Other

AUTHOR, DATE	SAMPLE INFORMATION (N, AGE RANGE/MEAN AGE + SD)	SES MEASURE	MODERATOR	OUTCOME	MAIN FINDINGS	KEY DIMENSION
**SHAHAEIAN ET AL. (2018)**	4,768, 4-5 years and 8-9 years	Composite SES	Shared book reading, receptive vocabulary	Early academic skills (4-5 years), academic achievement (8-9 years)	Shared reading at home was more strongly associated with academic achievement for children from lower SES and middle SES families compared to higher SES ones. The association of receptive language and writing and spelling scores was significantly larger in magnitude in the low SES group compared with the high SES group, and larger in magnitude in the middle SES group compared with the high SES group. The association between receptive language and mathematics achievement was significantly larger in magnitude in the middle SES group than the high SES group.	Stimulation
**XIA (2020)**	307, 5.53 years ± 0.50	Composite SES	Authoritative parenting style, authoritarian parenting style, permissive parenting style	School readiness (language and cognition)	The association of authoritative parenting with children's school readiness was significant for medium and low SES children but not for high SES children (high authoritative parenting buffered the association of low SES with low school readiness).	SSS

Search terms

TX (socioeconomic disadvantage OR socioeconomic status OR poverty OR neighborhood disadvantage OR neighbourhood disadvantage OR neighborhood SES OR neighbourhood SES OR SES OR neighborhood OR neighbourhood OR neighborhood adversity OR neighbourhood adversity OR household income OR parent income OR disadvantage OR socioeconomic factors OR Hollingshead OR parent education) AND TX (childhood or adolescent or youth or child or children or adolescence) AND TX (mediat* OR moderat* OR buffer OR mechanism OR intervention) AND TX (executive function or cognitive control or executive functioning or self-regulation or working memory or inhibition or inhibitory control or shifting or cognitive flexibility or attention or memory or language or verbal fluency or academic achievement or academic performance)

Quality assessment rating

Study name	Quality Rating
Agirdag (2018)	Good
Ainsworth (2002)	Fair
Albert et al. (2020)	Good
Altschul (2012)	Good
Bachman et al. (2022)	Good
Baker et al. (2018)	Good
Baker et al. (2020)	Fair
Barnes et al (2022)	Good
Barr (2015)	Good
Baydar & Akcinar (2015)	Fair
Beisly et al (2020)	Good
Bernardo et al. (2021)	Good
Betancourt et al. (2015)	Good
Betancur et al. (2018)	Good
Blakey et al. (2020)	Good
Bodovski & Farkas (2008)	Good
Boyes et al. (2017)	Good
Cadima et al. (2015)	Good
Callan et al. (2017)	Good
Carolan (2015)	Good

Cascella (2019)	Good
Cascella (2020)	Good
Chen at al. (2018)	Good
Cheng & Wu (2017)	Fair
Cheung & Wong (2020)	Good
Chevalère et al. (2022)	Fair
Chien & Mistry (2013)	Good
Claro et al. (2016)	Good
Coddington et al. (2014)	Fair
Coley et al. (2019)	Good
Crook & Evans (2014)	Good
Crosnoe & Cooper (2010)	Good
Cubides-Mateus et al. (2022)	Good
Daneri et al. (2018)	Good
Dearing et al. (2009)	Good
Dilworth-Bart (2012)	Fair
Dilworth-Bart et al. (2009)	Good
Dolean et al. (2019)	Good
Dulay et al. (2018)	Fair
Dupéré et al. (2010)	Good
Eamon (2002)	Good
Ellefson et al. (2020)	Good
Elliott & Bachman (2018)	Good
Encinger et al. (2020)	Good
Fagan (2016)	Good
Fekonja-Peklaj et al. (2015)	Fair
Fitzpatrick et al. (2014)	Good
Forget-Dubois et al. (2009)	Good
Foster et al. (2005)	Good
Fung & Chung (2019)	Good
Galindo & Sonnenschein (2015)	Good
Garrett-Peters et al. (2016)	Good
Geoffroy et al. (2007)	Good
Gonzalez et al. (2016)	Fair

Greenfader (2019)	Good
Hackman et al. (2015)	Good
Hamilton et al. (2016)	Good
Hartanto et al. (2018)	Good
He & Yin (2016)	Fair
Hoff (2003)	Fair
Iruka et al. (2014)	Fair
Kao et al. (2018)	Good
King & Trinidad (2021)	Good
Koepp et al. (2022)	Good
Kohen et al. (2008)	Good
Korzeniowski et al. (2016)	Fair
Kranjac & Kranjac (2021)	Good
Kriegbaum & Spinath (2016)	Good
Larson et al. (2015)	Good
Laurin et al. (2015)	Good
Lawson & Farah (2015)	Good
Lei (2018)	Good
Li et al. (2016)	Good
Lipina et al. (2013)	Fair
Little et al., (2018)	Good
Liu et al. (2015)	Good
Loboda et al. (2016)	Fair
Long & Pang (2016)	Fair
Losier et al. (2022)	Good
Luo et al. (2022)	Good
Lurie et al. (2021)	Good
Marks et al. (2006)	Fair
McCormick et al. (2017)	Good
McCoy, Conners et al. (2015)	Good
McCoy, Zuilkowski et al. (2015)	Fair
McNally et al. (2019)	Good
Mendive et al. (2016)	Fair
Merz et al. (2014)	Good

Ming et al. (2021)	Good
Mistry et al. (2004)	Good
Murphy et al. (2022)	Good
Myrberg & Rosén (2008)	Good
Myrberg & Rosen (2009)	Good
Natale et al. (2021)	Good
Nesbitt et al. (2013)	Good
Neuenschwander et al. (2017)	Good
Olsen & Huang (2021)	Good
Pearman (2020)	Good
Perry et al. (2017)	Good
Perry et al. (2018)	Good
Philbrook et al. 2017	Good
Piccolo et al. (2018)	Good
Raver et al. (2013)	Good
Raviv et al. (2004)	Good
Ren et al. (2020)	Good
Ribner et al. (2017)	Good
Richards et al. (2015)	Good
Rjosk et al. (2014)	Good
Rochette & Bernier (2014)	Fair
Rosen et al. (2019)	Good
Rubio-Codina et al. (2016)	Fair
Sarsour et al. (2011)	Fair
Schneider & Maguire (2022)	Good
Shahaeian et al. (2018)	Good
Singh et al. (2022)	Fair
Slusser et al. (2019)	Good
Speybroeck et al. (2012)	Fair
St. John & Tarullo (2019)	Fair
Sun et al. (2018)	Good
Suor et al. (2016)	Good
Swanson et al. (2019)	Good
Tomaszewski et al. (2020)	Good
Vogel et al. (2021)	Good

Vrantsidis et al. (2020)	Good
Waters et al. (2021)	Good
Wei et al. (2021)	Good
Wolf & McCoy (2017)	Good
Xia (2020)	Good
Xuan et al. (2019)	Good
Ye et al. (2021)	Good
Yeung et al. (2022)	Fair
Yu & Hannum (2007)	Good
Yu et al. (2020)	Good
Zhang et al. (2013)	Good
Zhang et al. (2019)	Good
Zhao et al. (2023)	Good
